# Dietary intervention improves metabolic levels in patients with type 2 diabetes through the gut microbiota: a systematic review and meta-analysis

**DOI:** 10.3389/fnut.2023.1243095

**Published:** 2024-01-08

**Authors:** Xiaoyu Xu, Fan Zhang, Jiajia Ren, Haimeng Zhang, Cuiqi Jing, Muhong Wei, Yuhong Jiang, Hong Xie

**Affiliations:** ^1^School of Public Health, Bengbu Medical University, Bengbu, China; ^2^Department of Epidemiology and Health Statistics, School of Public Health, Bengbu Medical University, Bengbu, China; ^3^Department of Nutrition and Food Hygiene, School of Public Health, Bengbu Medical University, Bengbu, China

**Keywords:** type 2 diabetes, dietary intervention, gut microbiota, short-chain fatty acids, systematic review

## Abstract

**Background:**

Poor dietary structure plays a pivotal role in the development and progression of type 2 diabetes and is closely associated with dysbiosis of the gut microbiota. Thus, the objective of this systematic review was to assess the impact of dietary interventions on improving gut microbiota and metabolic levels in patients with type 2 diabetes.

**Methods:**

We conducted a systematic review and meta-analysis following the PRISMA 2020 guidelines.

**Results:**

Twelve studies met the inclusion criteria. In comparison to baseline measurements, the high-fiber diet produced substantial reductions in FBG (mean difference −1.15 mmol/L; 95% CI, −2.24 to −0.05; I^2^ = 94%; *P* = 0.04), HbA1c (mean difference −0.99%; 95% CI, −1.93 to −0.03; I^2^ = 89%; *P* = 0.04), and total cholesterol (mean difference −0.95 mmol/L; 95% CI, −1.57 to −0.33; I^2^ = 77%; *P* = 0.003); the high–fat and low–carbohydrate diet led to a significant reduction in HbA1c (mean difference −0.98; 95% CI, −1.50 to −0.46; I^2^ = 0%; *P* = 0.0002). Within the experimental group (intervention diets), total cholesterol (mean difference −0.69 mmol/L; 95% CI, −1.27 to −0.10; I^2^ = 52%; *P* = 0.02) and LDL-C (mean difference −0.45 mmol/L; 95% CI, −0.68 to −0.22; I^2^ = 0%; *P* < 0.0001) experienced significant reductions in comparison to the control group (recommended diets for type 2 diabetes). However, no statistically significant differences emerged in the case of FBG, HbA1c, HOMA-IR, and HDL-C between the experimental and control groups. The high dietary fiber diet triggered an augmented presence of short-chain fatty acid-producing bacteria in the intestines of individuals with T2DM. In addition, the high-fat and low-carbohydrate diet resulted in a notable decrease in Bacteroides abundance while simultaneously increasing the relative abundance of Eubacterium. Compared to a specific dietary pattern, personalized diets appear to result in the production of a greater variety of beneficial bacteria in the gut, leading to more effective blood glucose control in T2D patients.

**Conclusion:**

Dietary interventions hold promise for enhancing metabolic profiles in individuals with T2D through modulation of the gut microbiota. Tailored dietary regimens appear to be more effective than standard diets in improving glucose metabolism. However, given the limited and highly heterogeneous nature of the current sample size, further well-designed and controlled intervention studies are warranted in the future.

## 1 Introduction

Type 2 diabetes mellitus (T2DM) is a non-infectious metabolic disease characterized by elevated blood glucose levels caused by impaired insulin secretion, insulin resistance, or both (1). The etiology of T2DM is multifaceted, and recent studies have identified the gut microbiota as a potential contributor to its pathophysiology (2). In addition to chronic inflammation and metabolic disturbances, individuals with T2DM experience dysregulation of the gut microbiota and compromised intestinal barrier function, leading to increased intestinal permeability (3). Specifically, gut microbiota imbalances manifest as reduced microbial abundance and diversity, particularly the depletion of butyric acid-producing bacteria (4). On one hand, patients exhibit diminished production of short-chain fatty acids (SCFAs) by beneficial gut bacteria, resulting in decreased levels of glucagon-like peptide-1 (GLP-1). On the other hand, the proliferation of harmful bacteria in the gut leads to increased intestinal permeability and the release of endotoxins (LPS), causing chronic inflammation in pancreatic islets, ultimately contributing to pancreatic β-cell damage and reduced insulin sensitivity (5, 6). Additionally, the gut microbiota is now recognized as a novel endocrine organ that influences human health by secreting metabolites such as SCFAs, bile acids, indoleacetic acid, branched-chain amino acids, and trimethylamine-N-oxide (7).

Furthermore, poor dietary structure and eating habits play significant roles in the onset and progression of T2DM, and an unbalanced diet is closely associated with gut microbiota dysbiosis. This dietary structure is characterized by chronic excessive consumption of carbohydrates and fats (particularly saturated fats) and inadequate intake of dietary fiber (8). Consequently, dietary interventions for patients with T2DM should focus on the overall dietary pattern rather than isolated nutrients or specific foods (9). This is because different dietary combinations and the nutrients contained within them interact synergistically to influence the health of individuals with T2DM (10).

While numerous meta-analyses and systematic reviews have examined the effects of dietary patterns or individual nutrients on glycemia in T2DM patients, few have explored the collective impact of daily dietary patterns on both gut microbiota and metabolic levels in this population. Hence, the objective of this systematic review is to investigate the efficacy of dietary patterns or combinations in improving gut microbiota and metabolic levels in patients with type 2 diabetes, while also exploring the potential mediating role of gut microbiota and its metabolites in the diet-metabolism relationship. Specifically, we will analyze changes in gut microbiota diversity, composition, and function. Probiotic interventions directly affecting the gut microbiota have been excluded from the inclusion criteria.

## 2 Materials and methods

This Meta-analysis and systematic review adhered to the latest PRISMA 2020 guidelines for reporting (11).

### 2.1 Eligibility criteria

The following criteria were used for inclusion and exclusion:

Experiment type: Population intervention trials such as randomized controlled trials. Experiments conducted on animals and *in vitro* were excluded.

Population: Inclusion encompassed patients diagnosed with type 2 diabetes or pre-diabetes, while exclusions were applied to individuals with type 1 diabetes, gestational diabetes, and other diabetes types. It was imperative for subjects with type 2 diabetes to possess a clinical diagnosis managed through dietary control, oral medications, and/or insulin therapy.

Intervention: Eligible interventions included specific dietary patterns or combinations of diets. Supplementation with nutrients, nutritional supplements, or probiotics in isolation was not considered for inclusion.

Outcomes: The primary outcomes were changes in the gut microbiota (including changes in the diversity and abundance of the gut microbiota, gut microbiota composition and gut microbiota function) and metabolic levels. Among the metabolic levels were blood glucose (glycated hemoglobin, fasting glucose, and insulin resistance homeostasis model assessment) and lipid profile (total cholesterol, triglycerides, LDL cholesterol, and HDL cholesterol). Secondary outcomes were changes in anthropometric indicators and inflammatory markers (endotoxin levels, tumor necrosis factor alpha, interleukin 6, and C-reactive protein).

### 2.2 Information sources and search strategy

Two authors independently searched electronic databases including PubMed, EMBASE, Web of Science, and ScienceDirect. The search was limited to articles published in English, with the completion date set as October 10, 2023. Medical subject headings (MeSH) and their synonyms were used as search terms, combined using Boolean operators (OR/AND) based on the study requirements. The search strategy is: “type 2 diabetes” combined with Boolean operator OR to similar terms (“type 2 diabetes mellitus”; “T2D”; “T2DM”; “pre-diabetes”; “prediabetes”; “prediabetic state”); AND “dietary pattern” combined with Boolean operator OR to similar terms (“feeding pattern”; “eating behavior”; “diet”; “dietary habit”; “food selection”); AND “gut microbiota” combined with Boolean operator OR to similar terms (“gut microbiome”; “gut microbiota”; “Intestinal flora”; “Gut microbiota”; AND “randomized controlled trial” combined with Boolean operator OR to similar terms (“RCT”; “trial”; “intervention”).

### 2.3 Selection process and data extraction

All titles and abstracts obtained from the literature search were initially screened manually, following the inclusion and exclusion criteria. Subsequently, the literature selected from the initial screening underwent a second screening through full-text reading. Finally, the studies identified in the secondary screening were organized using Microsoft Excel software. We extracted information from the included studies, including authors, country, year, diet and subgroup, study type, subject characteristics, and study outcomes (Table 1). The data extracted for analysis included relevant indicators such as baseline and post-dietary intervention measurements of gut microbiota, glucose, lipids, and inflammation.

**Table 1:** Description and characteristics of the included studies.

**References/** **Country**	**Dietary patterns and groupings**	**Nutritional characteristics of the intervention group**	**Study Design**	**Subject** **characteristics**	**Main microbiota results**	**Main clinical outcomes**
Candela et al. (12) Italy	Experimental group: fiber-rich longevity Ma-Pi 2 diet (*n* = 28) Control group: Italian Professional Association for T2D Therapy recommended diet CTR (*n* = 28) Healthy group: normal diet (*n* = 13)	High dietary fiber	Open randomized controlled trial, 21 days	T2DM, *n* = 56, BMI of 27–45 kg/m^2^, 55–70 years old; Healthy group: normal weight, 21-40 years old.	Both dietary interventions demonstrated effectiveness in alleviating gut microbiota dysbiosis and promoting the restoration of bacteria that produce short-chain fatty acids (SCFAs) in individuals with T2DM.	The reduction in HOMA-IR, total cholesterol and LDL/HDL ratio was significantly higher in the Ma-Pi 2 diet group than in the CTR group. the Ma-Pi 2 diet significantly reduced TNF-α, plasma CRP and IL-6 levels, while only TNF-α was significantly reduced in the CTR group.
Zhao et al. (13) China	Experimental group: high dietary fiber diet (*n* = 27) Control group: 2013 version of the Chinese Diabetes Association dietary guidelines for patients to manage their diet (*n* = 16)	High dietary fiber	Randomized controlled trial, 84 days	T2DM, *n* = 43, acarbose as a treatment drug	The high dietary fiber intervention increased 15 strains of acetate and butyric acid-producing bacteria, inhibited perindole- and hydrogen sulfide-producing bacteria, and promoted GLP-1 and PYY secretion to improve blood glucose, the abundance and diversity of which correlated significantly with clinical outcomes.	Indicators such as HbA1c improved faster and better in the experimental group than in the control group, and this clinical effect could be reproduced in mice by colony transplantation.
Chen et al. (14) China	Experimental group: high dietary fiber diet (*n* = 9) Control group: 2013 version of the Chinese Diabetes Association dietary guidelines for patients to manage their diet (*n* = 8)	High dietary fiber	Randomized controlled trial, 4 weeks	T2DM, *n* = 17, acarbose as a therapeutic agent	The ratio of *Firmicutes* to *Bacteroidota* was significantly lower in the treatment group, and the number of *Proteus* was reduced; the proportion of beneficial microorganisms of several genera increased, and the relative abundance of all other opportunistic pathogens decreased.	Glucose homeostasis, glucose homeostasis and systemic inflammation levels were significantly improved in the treatment group compared to the control group.
Medina-Vera et al. (15) Mexico	Experimental group: Functional food diet (*n* = 81) (T2DM) Control group: normal diet (healthy population)	High dietary fiber, low carb, high unsaturated fat	Randomized controlled trial, 12 weeks	T2DM, *n* = 81, 30-60 years old, and BMI of 18.5-24.9 Kg/m^2^ Healthy control group, 20-40 years old	Compared to the control group, the experimental group showed a significant increase in the α-diversity of the gut microbiota and significant changes in the abundance of specific flora, which were not associated with antidiabetic drugs. Among them, P Copri decreases, while *Faecalibacterium praussnitzii* and *Akkermansia* with anti-inflammatory effects increase.	The intervention group also had significantly lower blood glucose, total and LDL cholesterol, FFA, HbA1c, triglycerides and area under the CRP curve, and increased antioxidant activity compared to the control group.
Jian et al. (16) Finland and 8 other countries	Low energy diet for the first 8 weeks and weight maintenance for the last 148 weeks (*n* = 211)	Low-carbohydrate, low-fat	Multicenter randomized controlled trial, 3 years	Prediabetes overweight adult patients, *n* = 211, 25–70 years old, BMI ≥ 25 kg/m^2^	There was a significant increase in the relative abundance of several genera linked to enhanced metabolism. Changes in microbiota composition and predicted function were strongly correlated with weight loss. The initial characteristics of the gut microbiota accounted for approximately 25% of the variability in overall changes in adiposity prior to low-energy diet treatment.	Subjects lost an average of 11.5% of body weight and 22% of total body fat during the intervention, with significant improvements in all metabolic parameters. 76 subjects returned to normal blood glucose levels. Substantial interindividual variability was observed in the changes induced by the low-energy diet in variables related to glucose metabolism and total body fat.
Ismael et al. (17) Portugal	Mediterranean diet (*n* = 9)	High fiber and unsaturated fat	Single-arm trial, 12 weeks	T2DM, *n* = 9 (6 males, 3 females), 40–80 years old (mean 66 ± 9 years), except for 1 subject, all received oral hypoglycemic drugs	After 4 weeks, there was an increase in the abundance of intestinal bacteria, and the ratio of *Prevotella*/*Bacteroides* also increased. Bacterial diversity showed a negative correlation with HbA1c, while bacterial abundance exhibited negative correlations with FBS and HOMA-IR. Changes in gut microbiota seemed to precede alterations in a conventional biomarker for type 2 diabetes, namely HbA1c.	HbA1c and HOMA-IR were significantly reduced after 12 weeks. Blood lipid profiles showed no concomitant changes. Alkaline phosphatase activity (a marker of intestinal inflammation and permeability) in fecal samples was negatively correlated with HbA1c and positively correlated with bacterial diversity.
Deledda et al. (18) Italy	Experimental group: ketogenic diet (*n* = 6) Control group: Mediterranean diet (*n* = 5)	Very low-carb, high-fat	Randomized controlled trial, 12 weeks	T2DM newly diagnosed and without complications, *n* = 11 (6 males, 5 females), 45-65 years, BMI ≥ 28 Kg/m^2^	In the ketogenic diet group, there was a significant increase in beneficial microbiota groups, along with a decrease in microbiota groups associated with obesity (*Firmicutes* and Actinobacteriota) or other diseases. The Mediterranean diet group exhibited a significant increase in *Actinobacteria* and *Firmicutes*.	The beneficial effects of the ketogenic diet on anthropometric parameters were more significant than those of the Mediterranean diet, but there were no statistically significant differences in biochemical improvements. Macrogenomic alterations associated with certain metabolic pathways were found only in the ketogenic diet group.
Ren M, et al. (19) China,	Experimental group: almond-based low-carbohydrate diet a-LCD (*n* = 22) Control group: low-fat diet LFD (*n* = 23)	Low-carb, high-fat	Randomized controlled trial, 12 weeks	T2DM, *n* = 45, ≥18 years old	The consumption of a low-calorie diet (a-LCD) notably augmented the presence of short-chain fatty acid-producing bacteria, including *Roseburia, Ruminococcus*, and *Eubacterium*.	HbA1c levels were significantly lower during the study period in both groups compared to baseline. At Month 3, the a-LCD group had higher GLP-1 concentrations than the LFD group, had a greater decrease in HbA1c levels than the LFD, and significantly improved depressive symptoms.
Balfegó et al. (20) Spain	Experimental group: sardine diet (*n* = 19) Control group: general diet recommended for diabetes without sardines (*n* = 16)	High unsaturated fat	Randomized controlled trial, 6 months	T2DM, *n* = 35 (16 males, 19 females), BMI of 26–35 kg/m^2^, 40–70 years old, not receiving insulin and oral hypoglycemic drugs.	Both dietary interventions effectively lowered the concentrations of phylum *Firmicutes* and E. coli compared to their respective baselines. Moreover, the intervention group displayed a reduced *Firmicutes*/*Bacteroidetes* ratio and an increased abundance of *Bacteroides*-*Prevotella*.	There was no significant difference in glycemic control between the groups. Plasma insulin and HOMA-IR were reduced in both groups at 6 months after baseline. Plasma lipocalin increased only in the intervention group (+40.7%) compared to baseline levels. Omega-3 index increased by 2.6% in the experimental group and by 0.6% in the control group.
Karusheva et al. (21) Germany	Experimental group: reduced branched-chain amino acid diet (BCAA-) Control group: complete amino acid diet (BCAA+)	Reduction in branched-chain amino acids	Crossover test, 4 weeks	T2DM, *n* = 12, 40–60 years old, BMI of 28–35 kg/m^2^, disease duration < 5 years.	In comparison to the BCAA+ diet, the BCAA- diet intervention demonstrated an 11% decrease in the abundance of *Firmicutes* and a remarkable 40% increase in the abundance of *Bacteroidetes*.	After the BCAA-diet, insulin secretion was reduced, postprandial insulin sensitivity was increased, and mitochondrial efficiency in adipose tissue was stimulated.
Meleshko et al. (22) Ukraine	Experimental group: personalized diet (*n* = 35) Control group (*n* = 21)	NA	Randomized controlled trial, 18 days	T2DM, *n* = 56, 39–68 years old, all female.	Enterococcus faecalis, Escherichia coli, lac+, and Candida spp. significantly decreased, while *Lactobacillus* spp. significantly increased.	Significant improvements in blood glucose, lipid profile (cholesterol, LDL, HDL, VLDL, triglycerides) and inflammatory markers (IL-1 β, IL-10, IgA, TNF-α).
Shoer et al. (23) Israel	Experimental group: personalized diet (*n* = 100) Control group: Mediterranean diet (*n* = 100)	NA	Randomized controlled trial, 6 months	Prediabetes, *n* = 200, adults	The personalized diet had a greater effect on the gut microbiota than the Mediterranean diet. The personalized diet resulted in a significant increase in the relative abundance and alpha diversity of 19 gut microbiota species. flavonifractor plautii, Roseburia hominis, Ruthenibacterium lactatiformans and Faecalibacterium prausnitzii increased significantly in abundance. The Mediterranean diet resulted in a significant increase in the relative abundance of four gut microbiota species.	Compared to the Mediterranean diet, the personalized diet had a greater effect on glycemic control (HbA1c).

FBS, fasting blood sugar; HbA1c, glycated hemoglobin; HOMA-IR, homeostasis model assessment of insulin resistance; FFA, free fatty acids; plasma CRP, C-reactive protein; IL-6, interleukin; Functional food diet: Rich in soluble fiber, prebiotics, plant protein, and n-3 unsaturated fatty acids; Personalized diet: Using developed algorithms, personalized diets are selected based on the patient's gut microbiota, immune, and biochemical parameters.

### 2.4 Data analysis

Meta-analysis was conducted using Review Manager 5.4 software. All included studies were randomized controlled trials, except for the study by Ismael S et al., which was a single-arm trial with only one experimental group in its study subgroup. In our meta-analysis, two distinct approaches were employed. Firstly, we conducted a comparative assessment between the changes observed in the experimental group pre- and post-intervention and those in the control group (following a recommended diet for patients with type 2 diabetes mellitus) before and after their respective interventions. Secondly, we compared changes before and after the intervention of two nutritionally characterized diets (high-fiber diets, high-fat and low-carbohydrate diets) within groups. The specific indicators scrutinized encompassed alterations in fasting blood glucose, glycated hemoglobin, the homeostasis model insulin resistance index, total cholesterol, triglycerides, LDL cholesterol, HDL cholesterol, and body mass index. The mean and standard deviation were calculated using the conversion formula in an Excel sheet (24, 25), and the standard error was converted to standard deviation using a specific formula. Fasting glucose units were standardized to mmol/L.

### 2.5 Risk of bias and quality assessment

Review Manager 5.4 software was used as an assessment tool for evaluating the quality of clinical trial studies. Two authors independently assessed the risk of bias and quality of each included article. Areas assessed included selection bias (random sequence generation and allocation concealment), implementation bias (blinding of participants and personnel), measurement bias (blinding of outcome assessment), missing visit bias (incomplete outcome data), reporting bias (selective reporting), and other biases. Each assessment was categorized as “low risk,” “high risk,” or “uncertain risk.”

## 3 Description and classification of dietary interventions

In light of the intricate and multifarious nature of various dietary patterns, this study categorizes them into two distinct classes: specific dietary patterns and individualized dietary patterns, contingent upon whether the groups underwent uniform dietary interventions. Furthermore, within the category of specific dietary patterns, a finer classification was employed to delineate high-fiber diets, high-fat and low-carbohydrate diets, and low-fat low-carbohydrate diets, guided by the nutritional characteristics of the dietary interventions encompassed. Table 2 describes the characteristics of the main nutrients in dietary interventions.

**Table 2:** The characteristics of the main nutrients in dietary intervention.

**Categorization of dietary patterns**	**Dietary intervention measures**	**Specific dietary characteristics**	**Main nutrient characteristics**			
			**Dietary fiber**	**Carbohydrate**	**Lipid**	**Protein**
Specific dietary patterns	Fiber-rich longevity Ma-Pi 2 diet (12)	High dietary fiber; Rich in vegetables, fruits, grains, and white meat; No added sugar.	High-fiber	NA	Low saturated fat	NA
	High dietary fiber diet (13)	High dietary fiber; Rich in whole grains and prebiotics.	High-fiber	NA	NA	NA
	High dietary fiber diet (14)	A high fiber diet composed of whole grains and prebiotics.	High-fiber	NA	NA	NA
	Functional food diet (15)	Rich in soluble fiber, prebiotics, plant protein, and n-3 unsaturated fatty acids.	High-fiber	Low carbohydrate	High unsaturated fat	NA
	Mediterranean diet (17)	Rich in fiber, unsaturated fatty acids, and phytochemicals; Very low red meat and processed foods.	High-fiber	NA	High unsaturated fat	NA
	ketogenic diet (18)	Extremely low in carbohydrates and high in fat.	NA	Low carbohydrate	High fat	NA
	Mediterranean diet (18)	Rich in fiber, unsaturated fatty acids, and phytochemicals; Very low red meat and processed foods.	High-fiber	NA	High unsaturated fat	NA
	Low energy diet (16)	8 weeks of full meal replacement diet, followed by a low calorie diet for the next 148 weeks.	NA	Low carbohydrate	Low fat	NA
	Almond-based low-carbohydrate diet (19)	A large amount of nuts, low-carbon water, and high fat.	NA	Low carbohydrate	High fat	NA
	Low-fat diet (19)	Low fat.	NA	NA	Low fat	NA
	Sardine diet (20)	Take 100 g of sardine 5 days a week.	NA	NA	High unsaturated fat	NA
	BCAA- diet (21)	Reduce branched chain amino acids in dietary protein.	NA	NA	NA	Reduce branched chain amino acids
Personalized dietary patterns	Personalized diet (22)	Using developed algorithms, select personalized diets based on the patient's gut microbiota, immune, and biochemical parameters.	NA	NA	NA	NA
	Personalized diet (23)	Using developed algorithms, select personalized diets based on the patient's gut microbiota, immune, and biochemical parameters.	NA	NA	NA	NA

NA, Not Applicable.

## 4 Results

### 4.1 Study selection

**Figure 1:**
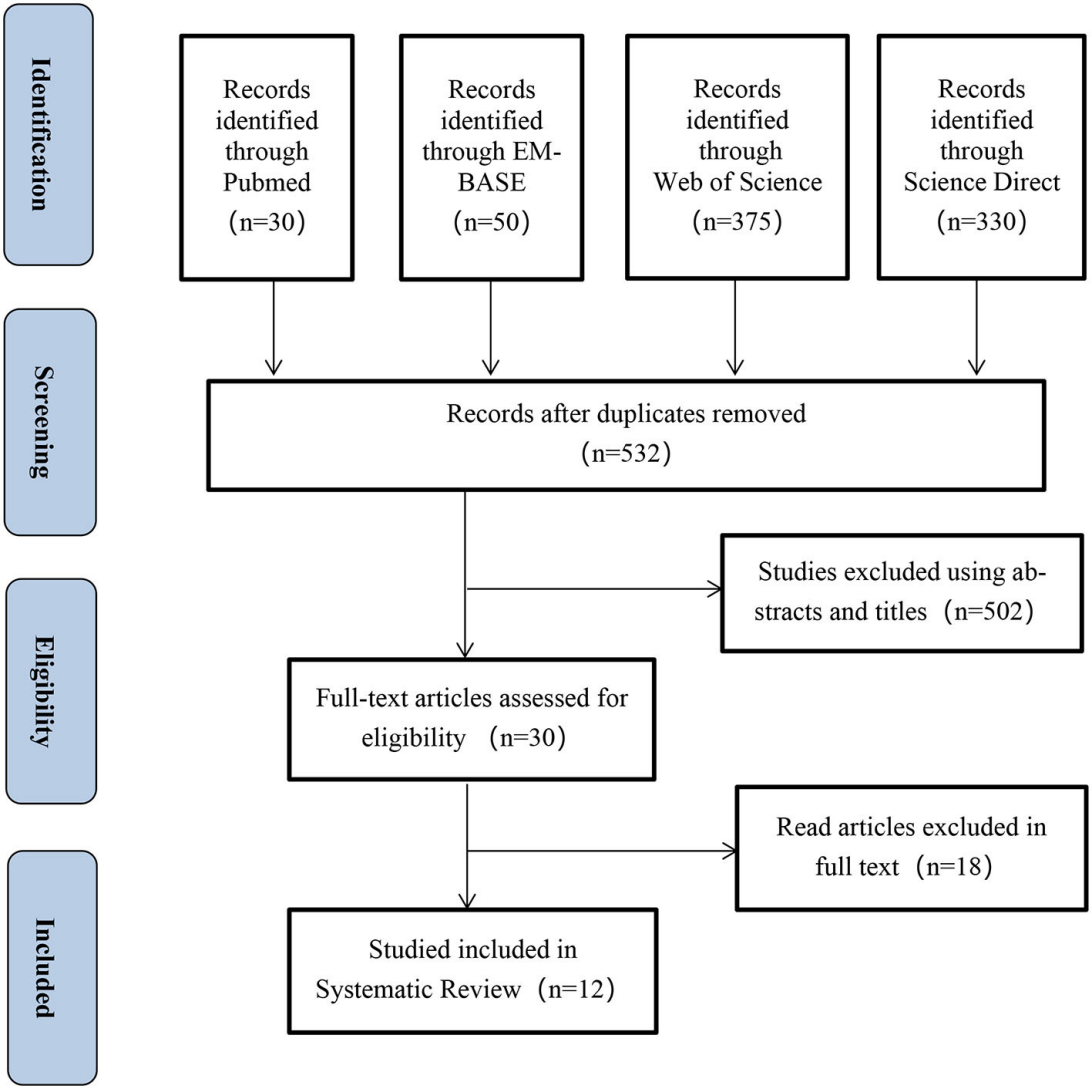
PRISMA flowchart.

Using the literature search strategy, we initially identified 785 relevant citations from four databases: Pubmed, EMBASE, Web of Science, and Science Direct. After removing duplicate citations, 532citations underwent title and abstract screening based on the inclusion and exclusion criteria, resulting in the exclusion of 502 citations. Finally, 12clinical studies were included in the meta-analysis after a thorough evaluation of the full text to ensure they met the specified study types, interventions, and study outcomes. Figure 1 presents the PRISMA flow chart illustrating the selection process of the included studies.

### 4.2 Study characteristics

#### 4.2.1 Participant characteristics

The study involved a total of 676 individuals with either type 2 diabetes mellitus (T2DM) or prediabetes, ranging in age from 25 to 80 years. One study reported a BMI of 18.5–24.9 kg/m^2^ (15), while 6 studies provided a BMI range of 25–45 kg/m^2^, and the remaining five did not report BMI values. These findings indicate that at least half of the participants were overweight or obese.

#### 4.2.2 Intervention characteristics

The duration of the interventions ranged from 18 days to 3 years. The interventions consisted of various dietary patterns or combinations, including 3 Mediterranean diets (Two of them were compared with ketogenic diet and personalized diet, respectively), 1 low-energy diets, 1 low-carbohydrate diet based on almonds, 1 low-fat diet, 1 Ma-Pi 2 diet, 2 high dietary fiber diets, 1 diet incorporating sardines, 1 diet with reduced branched-chain amino acids, and 2 individualized diet. Supplementary Table S1 provides details of the nutritional intake for each experimental group after the dietary intervention, as anticipated in the experiment.

### 4.3 Risk of bias in studies

We assessed the quality of the 11 included studies using the Cochrane Collaboration Risk of Bias tool (Supplementary Figure S1) (26). All studies were determined to have low risk of bias in terms of missing visit bias (incomplete outcome data) and reporting bias (selective reporting). Only three studies (13, 19, 20) explicitly mentioned using random sequence generation for allocation, and two of these studies (13, 19) also reported concealing the allocation scheme, indicating low risk of selection bias. Implementation bias (blinding of participants and personnel) and measurement bias (blinding of outcome assessment) were rated as lower in four studies, while two studies exhibited a higher risk of bias associated with blinding of participants and personnel (14, 18).

### 4.4 Effect of dietary intervention on gut microbiota

#### 4.4.1 Changes in gut microbiota diversity and richness

Five studies investigated gut microbiota diversity, while two studies examined gut bacterial richness. In a multicenter randomized clinical trial (16) involving 211 pre-diabetic overweight adult patients, a low-energy diet intervention resulted in significant increases in alpha diversity (microbiota composition diversity within individuals), beta diversity (differences in microbiota structure between individuals), and gut microbiota richness (all *P* < 0.001). Ren et al. (19) observed increased alpha diversity of the gut microbiota in patients with T2DM following both an almond-based low-carbohydrate diet and a low-fat diet, although there was no significant difference in gut microbiota structure (beta diversity) between the two diet groups. Candela M et al. (12) compared the fiber-rich long-life Ma-Pi 2 diet with the Italian T2D Therapy Professional Association recommended diet and found a tendency for both dietary patterns to increase gut microbiota alpha diversity in T2DM patients, but without significant differences between time points.

Ismael et al. (17) conducted a single-arm trial of a Mediterranean diet intervention, where no difference in gut microbiota diversity was observed at the end of the intervention, while bacterial richness tended to increase from baseline to 12 weeks. However, Deledda et al. (18) also implemented a Mediterranean diet intervention, comparing it with a ketogenic diet. The results indicated no significant difference in the alpha diversity of the gut microbiota over time in either group, but a significant difference in beta diversity of the flora between the two intervention groups (*p* = 0.013).

#### 4.4.2 Changes in the composition of the gut microbiota

Table 3 displays the effects of different dietary interventions on the gut microbiota at the phylum, genus, and species levels. Candela et al. (12) reported that a fiber-rich Ma-Pi 2 diet effectively countered the reduction of *Faecalibacterium, Bacteroides*, and *Dorea* in T2DM patients and promoted the presence of SCFA-producing bacteria, such as *Faecalibacterium prausnitzii* and *Lachnospiraceae bacterium*. Zhao et al. (13) demonstrated that a high dietary fiber intake stimulated the production of 15 strains of acetate and butyrate-producing bacteria, while inhibiting the production of indole and hydrogen sulfide-producing bacteria. Chen et al. (14) found that a high dietary fiber intake increased the proportion of several beneficial bacteria in the intestines of T2DM patients, while decreasing the proportion of certain opportunistic pathogenic bacteria.

**Table 3:** Effect of different diets on part of the gut microbiota in T2DM patients at Phylum, Genus and Species levels.

**Phylum**	**Genus**	**Ma-Pi 2 diet (12)**	**High fiber diet (13)**	**High fiber diet (14)**	**Functional food diet (15)**	**Low energy18mm diet (16)**	**Mediterranean diet (17)**	**Ketogenic diet (18)**	**Mediterranean diet (18)**	**Low Carbohydrate diet (19)**	**Low-fat diet (19)**	**Sardine diet (20)**	**BCAA+ and BCAA- (21)**	**Personalized diet (22)**	**Personalized diet (23)**
*Bacteroidetes*		NR	NR	NR	NR	↑^***^	NR	↓	↓^*^	↓^*^	↓	↓	BCAA- has a 40% higher abundance of *Bacteroidetes* than BCAA+.	NR	NR
	*Bacteroides*	↑	NR	↑	*Bacteroides* fragilis↑	↑^***^	↑	↓^*^	↓^*^	↓^*^	↓	NR	NR	NR	NR
	*Prevotella*	NR	NR	↓	*Prevotella copri* reduced by 13%↓	NR	NR	NR	NR	NR	NR	↑	NR	NR	NR
*Firmicutes*		NR	NR	NR	NR	↓^***^	↓	↓^*^	↑^*^	NR	NR	↓^**^	The abundance of *Firmicutes* in BCAA - is 11% lower than that in BCAA+.	NR	NR
	*Ruminococcus*	↓	NR	↑	NR	↑^***^	↑	↓^*^	NR	↑	↓^*^	NR	NR	NR	*Ruthenibacterium lactatiformans*↑^*^
	*Faecalibacterium*	↑	NR	NR	NR	↓^***^	↓	NR	NR	NR	NR	NR	NR	NR	NR
	*Roseburia*	↑	NR	NR	NR	NR	↑	NR	NR	↑^*^	↓^**^	NR	NR	NR	Roseburia hominis↑^*^
	*Clostridiumleptum*	NR	*Faecalibacterium prausnitzii*↑	NR	*Faecalibacterium prausnitzii*↑	NR	NR	NR	NR	NR	NR	*Faecalibacterium prausnitzii*↑	NR	NR	*Faecalibacterium prausnitzii*↑^*^
	*Eubacteriu*	NR	↑	NR	NR	NR	NR	↑^*^	NR	↑^**^	↑	NR	NR	NR	NR
	*Lachnospir*	↑	*Lachnospira*ceae bacterium↑	NR	NR	↓^***^	NR	NR	NR	NR	NR	NR	NR	NR	NR
	*Pseudobutyrivibrio*	NR	NR	NR	NR	↓^***^	NR	NR	NR	NR	NR	NR	NR	NR	NR
	*Lactobacillus*	NR	NR	NR	NR	NR	NR	NR	NR	NR	↓	NR	NR	↑^*^	NR
*Verrucomicrobia*	*Akkermansia*	↑	NR	NR	*A.Muciniphila* increased by 125% ↑	↑^***^	↑	↑^*^	NR	NR	NR	NR	NR	NR	NR
*Actinobacteria*		NR	NR	NR	NR	↓^***^	↑	↓^*^	↑^*^	NR	NR	NR	NR	NR	NR
	*Bifidobacterium*	NR	↑	↑	*Bifidobacterium* longum↑	↓^***^	NR	NR	NR	NR	NR	NR	NR	NR	NR

All studies are within-group comparisons except for BCAA+ vs. BCAA- for between-group comparisons: ↑indicates increased bacterial abundance compared to baseline, ↓indicates decreased bacterial abundance compared to baseline. In studies where p-value sizes are provided, ^*^indicates p ≤ 0.05 compared to baseline, ^**^indicates p ≤ 0.01 compared to baseline, and ^***^indicates p ≤ 0.001 compared to baseline. indicates butyric acid-producing bacteria. NR, Not reported.

Medina-Vera et al. (15) observed that a Functional food diet increased the levels of *Akkermansia muciniphila* and *Faecalibacterium prausnitzii* (associated with anti-inflammatory effects) by 125%, while decreasing the levels of *Prevotella copri* by 13%. Additionally, the intake of *Bifidobacterium* longum (linked to improved insulin signaling) and *Bacteroides* fragilis (with a robust capacity for multiple dietary polysaccharides) also increased. Jian et al. (16) demonstrated that after 8 weeks of a low-energy diet intervention, there was a significant increase in the abundance of *Verrucomicrobia* and *Bacteroidetes* (*P* < 0.001) at the phylum level, while *Actinobacteria* and *Firmicutes* significantly decreased in abundance (*P* < 0.001). At the genus level, *Akkermansia, Ruminococcus, Bacteroides*, and *Christensenellaceae R-7* showed significant increases, whereas *Faecalibacterium, Bifidobacterium*, and butyrate-producing bacteria (*Lachnospira, Pseudobutyrivibrio*, and *Blautia*) were significantly reduced.

Ismael et al. (17) compared changes in *Bacteroides* flora at 4 and 12 weeks of Mediterranean diet intervention with baseline. The ratio of *Prevotella* to *Bacteroides* significantly increased after 4 weeks, and the increase in the ratio of *Firmicutes* to *Bacteroidetes* after 12 weeks was clinically significant. The relative abundance of *Bacteroides, Ruminococcus, Roseburia, Akkermansia*, and *Actinobacteria* showed an increasing trend after 12 weeks of Mediterranean diet intervention, while *Faecalibacterium* and *Firmicutes* exhibited a decreasing trend. Deledda et al. (18) divided the ketogenic and Mediterranean diets into two intervention groups, and both groups showed consistent reductions in *Bacteroidetes* and *Bacteroides*. The ketogenic diet group exhibited a significant increase in beneficial microbiota such as *Akkermansia* and *Eubacterium*, as well as a decrease in microbial taxa associated with obesity (*Firmicutes* and *Actinobacteria*). The Mediterranean diet group showed significant increases in *Firmicutes* and *Actinobacteria*. Ren et al. (19) compared an almond-based low-carbohydrate diet with a low-fat diet. After 3 months of intervention, the low-carbohydrate diet group exhibited significantly higher relative abundances of *Ruminococcus* and *Roseburia* (*P* < 0.01) compared to the low-fat diet group. Several short-chain fatty acid-producing bacteria, including *Eubacterium*, were significantly increased in the low-carbohydrate diet group compared to baseline.

Karusheva et al. (21) found an 11% decrease in the abundance of *Firmicutes* and a 40% increase in the abundance of *Bacteroidetes* after BCAA- intervention compared to BCAA+. Meleshko et al. (22) reported that the use of a personalized diet resulted in a significant increase in the abundance of *Lactobacillus* spp., Enterococcus faecalis, Escherichia coli, lac+, and Candida spp., while there was a significant decrease in abundance. In a study by Shoer et al. (23), it was observed that personalized dietary regimens elicited a more pronounced influence on gut microbiota in comparison to the Mediterranean diet. The personalized diet instigated a noteworthy rise in the relative abundance of 19 gut microbiota species, notably including Flavonifractor plautii, Roseburia hominis, Ruthenibacterium lactatiformans, and Faecalibacterium prausnitzii. In contrast, the Mediterranean diet led to a notable increase in the relative abundance of only four gut microbiota species.

Upon comprehensive analysis of alterations in gut microbiota composition across the included studies, it became evident that high dietary fiber-based diets (such as the Ma-Pi 2 diet, high dietary fiber diet, functional food diet, and Mediterranean diet) conferred an augmented presence of intestinal Bacteroides, Faecalibacterium prausnitzii, Akkermansia, and Bifidobacterium in patients with T2DM. In contrast, high-fat low-carbohydrate diets, encompassing ketogenic diets and almond-based low-carbohydrate diets, markedly diminished the relative abundance of Bacteroides and substantially augmented that of Eubacterium.

#### 4.4.3 Changes in gut microbiota function

Three studies conducted comparative predictive analyses of the functional macrogenome of the gut microbiota following dietary interventions, and some of the metabolic pathways that were significantly altered are collated in Supplementary Table S2 in the Appendix. Deledda et al. (18) reported a significant increase in 22 metabolic pathways commonly associated with the ketogenic diet group, while 17 pathways showed a significant decrease at months 2 and 3 compared to baseline. Notably, pathways involved in the degradation of limonene and ethylbenzene, biosynthesis of cephalosporin and penicillin, and carbohydrate digestion and absorption exhibited strong negative correlations, but displayed strong positive correlations with steroid and carotenoid biosynthesis as well as non-homologous end-joining pathways. No significant correlations with metabolic pathways were observed in the Mediterranean diet group.

Jian et al. (16) discovered that low-energy dietary intake significantly increased the abundance of *Akkermansia* (which promotes the glycosaminoglycan degradation pathway) and decreased the abundance of *Pseudobutyrivibrio* (which promotes flagellar assembly). Furthermore, the body mass index (BMI) and body weight of the subjects exhibited a negative correlation with the glycosaminoglycan degradation pathway and a positive correlation with flagellar assembly, indicating a connection between changes in the human gut microbiota and body weight.

Candela et al. (12) demonstrated that the Ma-Pi 2 diet reduced the abundance of gut microbiota marker bacteria associated with type 2 diabetes mellitus (involved in polyketide biosynthesis, sphingolipid biosynthesis, arachidonic acid metabolism, and alanine metabolism), while increasing the abundance of bacteria that improved metabolism (involved in taurine, cysteine, methionine, valine, leucine, isoleucine metabolism, and unsaturated fatty acid biosynthesis). Consequently, the Ma-Pi 2 diet provided the body with additional essential amino acids and vital nutrients.

### 4.5 Effect of dietary intervention on glycemic control

Candela et al. (12) compared the fiber-rich Ma-Pi 2 diet with the diet recommended by the Italian Professional Association for the Treatment of T2DM. Both diets led to a significant reduction in fasting blood glucose (FBG) levels in patients. The reduction in FBG (*P* = 0.007) and homeostatic model assessment of insulin resistance (HOMA-IR) (*P* = 0.0004) was more pronounced in the Ma-Pi 2 diet group compared to the control group. Zhao et al. (13) observed a significant decrease in both glycated hemoglobin (HbA1c) and FBG levels in the high dietary fiber group (*P* < 0.001) and the control group (following the 2013 edition of the Dietary Guidelines for Patients with Chinese Diabetes Society) (*P* < 0.001). However, the high dietary fiber group exhibited a greater reduction in HbA1c levels starting from day 28 of the intervention (−1.91±0.24). Chen et al. (14) found that high dietary fiber significantly lowered HbA1c and FBG levels in patients.

Medina-Vera et al. (15) reported significant reductions of −15.6% in free fatty acids (FFA) and −7.2% in HbA1c levels among patients following a high fiber low-energy diet intervention compared to baseline. Jian C et al. (16) observed significant reductions in HbA1c, FBG, and HOMA-IR in patients with T2DM after a low-energy diet (*P* < 0.001). Ren M et al. (19) compared low-carbohydrate and low-fat diets and found that the low-carbohydrate group exhibited a greater decrease in HbA1c levels compared to the low-fat diet group after 3 months of intervention (*P* < 0.01). Both groups demonstrated significantly lower HbA1c levels during the intervention (*P* < 0.01 and *P* < 0.05, respectively).

Ismael et al. (17) implemented a 12-week Mediterranean diet intervention in T2DM patients, resulting in a significant decrease in HOMA-IR (mean change −1.03±2.64, *P* < 0.05, Cohen's d = −0.41) and HbA1c compared to baseline levels (mean change −0.67±0.98, *p* < 0.05, Cohen's d = −0.70), although the decrease in FBG was not significant. Deledda et al. (18) demonstrated a 1.1% decrease in HbA1c in the ketogenic diet group after dietary intervention (from 6.6 ± 0.9 to 5.5 ± 0.5, *p* = 0.012), while the change in HbA1c in the Mediterranean diet group was not significant. No significant changes in FBG were observed in either group.

Balfegó et al. (20) found no significant difference in glycemic control between the sardine diet and the control diet. Both groups exhibited significantly lower homeostatic model assessment of insulin resistance (HOMA-IR) and fasting insulin levels compared to baseline, but the reduction was greater in the sardine diet group (mean change in fasting insulin −6.1 ± 1.8 mU/L, P=0.01; mean change in HOMA-IR −2.3 ± 0.7, *P* = 0.007). Karusheva et al. (21) demonstrated that a reduced branched-chain amino acid diet (BCAA-) led to reduced insulin secretion and increased postprandial insulin sensitivity when compared to a full amino acid diet (BCAA+). Meleshko et al. reported a significant reduction in blood glucose levels in patients with T2DM after personalized dietary intervention (mean change −2.36 ± 2.13 mmol/L, *P* < 0.05). Shoer et al. (23) reported that a personalized diet was more effective in managing glycemic control (HbA1c) compared to the Mediterranean diet.

**Figure 2:**
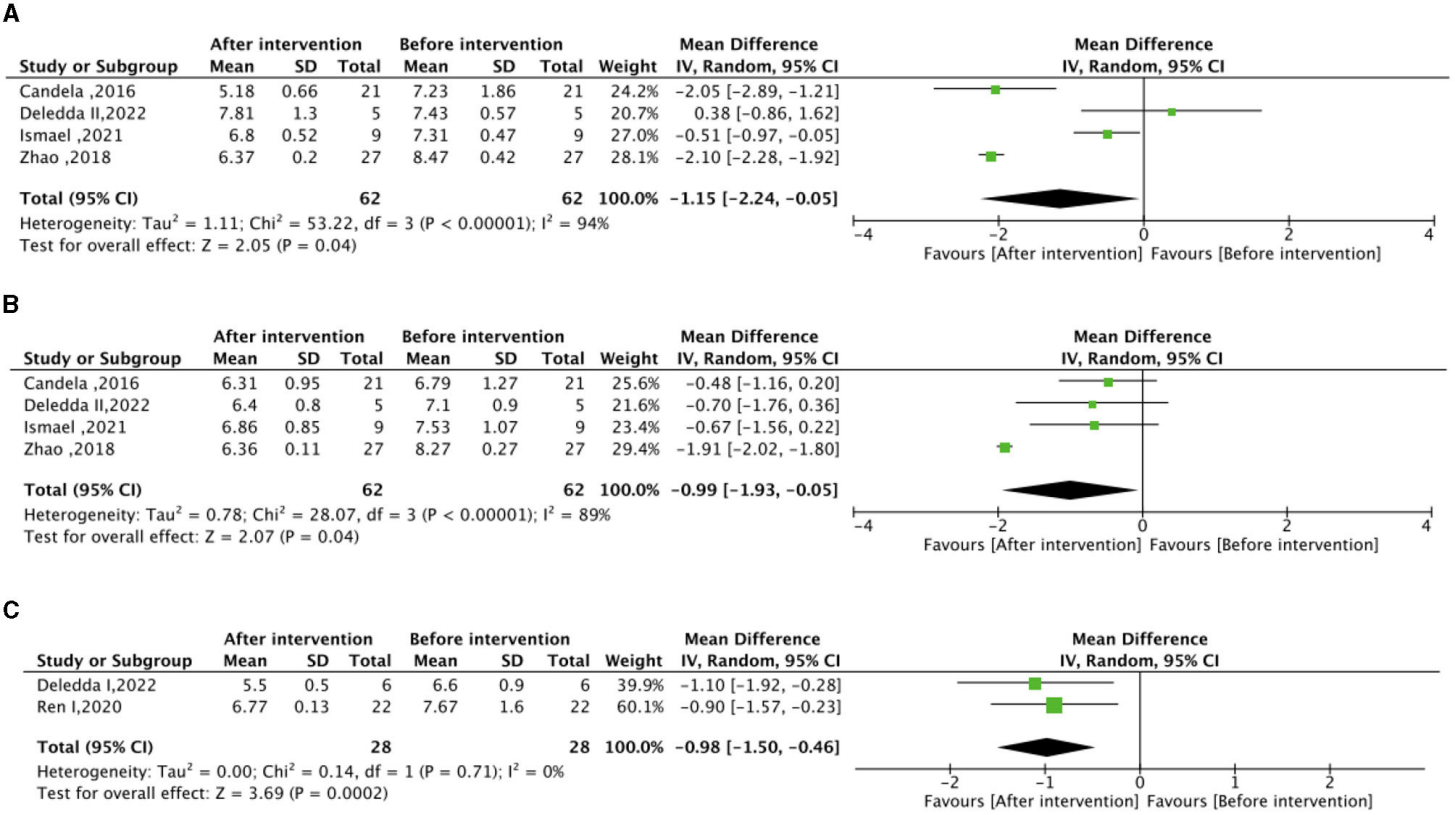
Effect of dietary intervention on glycemic control. **(A)** Own control of the effect of high dietary fiber dietary intervention on fasting blood glucose (FBG) (mmol/L). **(B)** Own control of the effect of high dietary fiber dietary intervention on glycated hemoglobin (HbA1c) (%). **(C)** Own control for the effect of high-fat low-carbohydrate dietary intervention on (HbA1c) (%).

Regarding the meta-analysis of glucose metabolism, no significant differences were observed in FBG, HbA1c, and HOMA-IR between the experimental group (intervention diet) and the control group (recommended diet for type 2 diabetic patients), as depicted in Supplementary Figure S2. Following the high-fiber dietary intervention, FBG (mean difference −1.15 mmol/L; 95% CI, −2.24 to −0.05; I^2^ = 94%; *P* = 0.04) and HbA1c (mean difference −0.99%; 95% CI, −1.93 to −0.03; I^2^ = 89%; P=0.04) exhibited significant reductions compared to baseline levels (Figure 2). Moreover, the high–fat low–carbohydrate HbA1c (mean difference −0.98; 95% CI, −1.50 to −0.46; I^2^ = 0%; *P* = 0.0002) was notably lower after the dietary intervention (Figure 2).

### 4.6 Effect of dietary intervention on lipids

Medina-Vera et al. (15) observed a decrease in total cholesterol (−7.8%), triglycerides (−23%), and LDL-C (−9.9%) compared to baseline values in 81 subjects who received a high fiber, low-energy diet. Additionally, Candela et al. (12) reported a significantly greater reduction in total cholesterol, HDL-C, and LDL-C in the Ma-Pi 2 diet group compared to the control group (*p* < 0.05).

**Figure 3:**
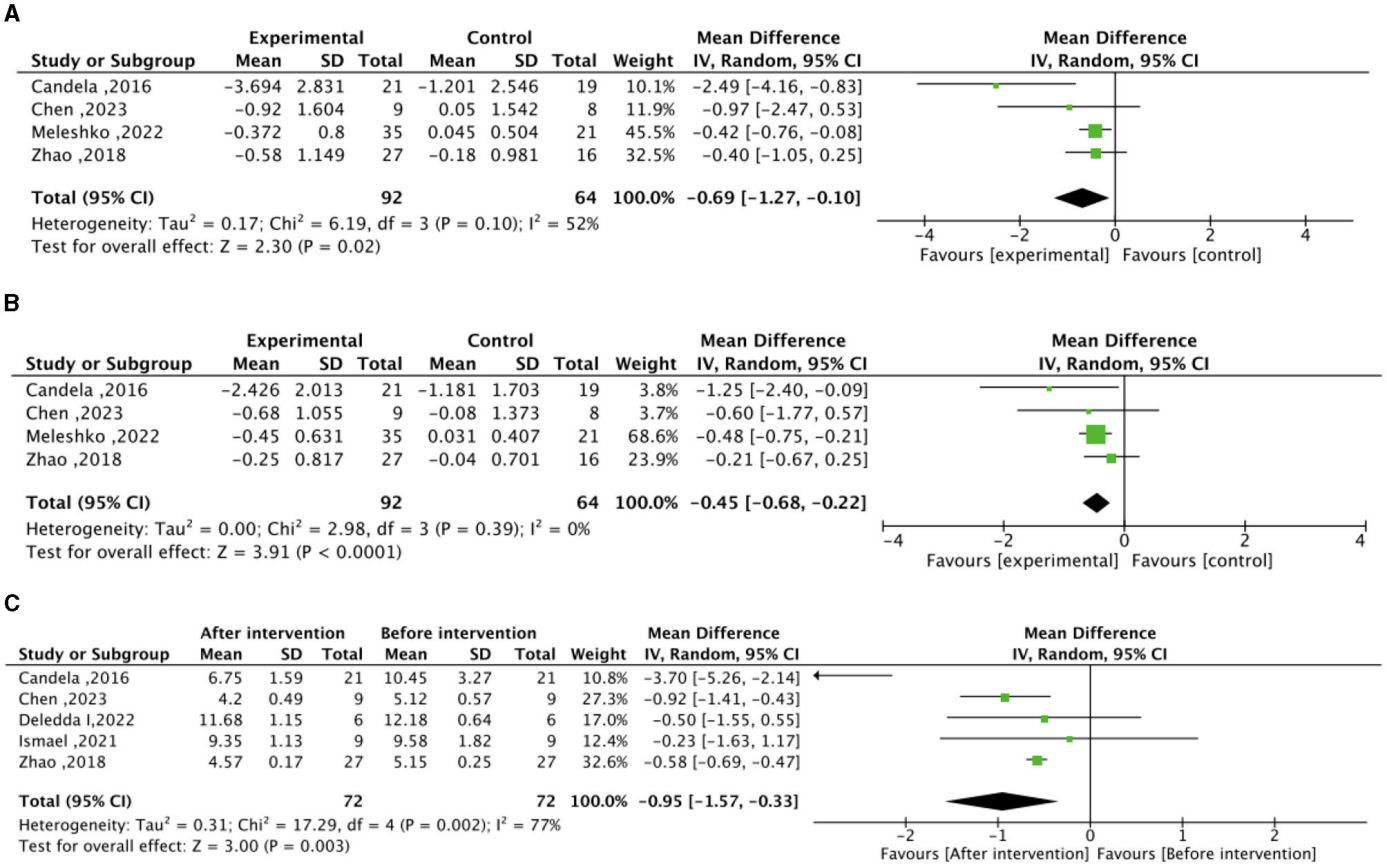
Effect of dietary intervention on lipid levels. **(A)** Effect of dietary intervention on total cholesterol (mmol/L) in the experimental group compared to the recommended diet for type 2 diabetic patients. **(B)** Effect of dietary intervention on low-density lipoprotein cholesterol (LDL-C) (mmol/L) in the experimental group compared to the recommended diet for type 2 diabetic patients. **(C)** Self-control of the effect of high dietary fiber dietary intervention on total cholesterol (mmol/L).

In the context of the meta-analysis on lipid metabolism, the experimental group (intervention diet) exhibited a noteworthy reduction in total cholesterol (mean difference −0.69 mmol/L; 95% CI, −1.27 to −0.10; I^2^=52%; P=0.02) and LDL–C (mean difference −0.45 mmol/L; 95% CI, – 0.68 to −0.22; I^2^=0%; *P* < 0.0001) compared to the control group (recommended diet for type 2 diabetic patients), as illustrated in Figure 3. After the high–fiber dietary intervention, total cholesterol (mean difference −0.95 mmol/L; 95% CI, −1.57 to −0.33; I^2^=77%; *P* = 0.003) demonstrated a significant decrease relative to baseline levels. Nonetheless, no significant differences were observed in HDL-C between the experimental and control groups, nor in the changes before and after the high-fiber diet intervention, as depicted in Supplementary Figure S2.

### 4.7 Effect of dietary intervention on inflammatory indicators

According to Candela et al. (12), the consumption of a high-fiber Ma-Pi 2 diet significantly reduced the levels of TNF-α (-18.63 ± 27.59 pg/mL), CRP (−4.43±7.81 mg/L), and IL-6 (−0.286±3.86 pg/mL) (*p* < 0.01). Medina-Vera et al. (15) demonstrated that a low-energy diet high in fiber, polyphenols, and plant proteins effectively reduced inflammation levels in T2DM patients. The intervention group showed a 65% reduction in endotoxin (LPS) levels compared to baseline. Similarly, Chen et al. (14) found that a high dietary fiber diet significantly decreased IL-6, IL-1, and TNF-α, resulting in reduced systemic inflammation. Meleshko et al. (22) designed a personalized diet that significantly reduced TNF-α levels (−6.9 ± 0.91 pg/mL, *P* < 0.05), along with IL-6 and IL-1, indicating the efficacy of dietary interventions rich in dietary fiber and phytochemicals in improving inflammation levels in T2DM.

### 4.8 Effect of dietary intervention on anthropometrics

Deledda et al. investigated the impact of the Mediterranean diet intervention, which resulted in significant reductions in weight (−3.1 ± 17.6 kg, *P* = 0.02), body mass index (−2.1 ± 4.53 kg/m^2^, *P* = 0.02), and waist circumference (−4.7 ± 9.08 cm, *P* = 0.004) compared to baseline. However, the ketogenic diet intervention yielded even more substantial and significant reductions in weight (−14.3 ± 13.91 kg, *P* < 0.0001), body mass index (−5.3 ± 3.93 kg/m^2^, *P* < 0.0001), and waist circumference (−12.3 ± 7.27 cm, *P* < 0.0002). The low–energy diet designed by Jian C et al. demonstrated significant decreases in BMI compared to baseline (−3.9 ± 1.14 kg/m^2^, *P* < 0.001), as did the low–carbohydrate diet implemented by Ren et al. (−0.51 ± 2.39 kg/m^2^, *P* = 0.034). Similarly, the individualized diet by Meleshko et al. led to a significant reduction in BMI during the intervention period (−4.03 ± 10.62 kg/m^2^).

Regarding the meta-analysis of BMI, no significant disparity in BMI was observed between the experimental group (intervention diet) and the control group (recommended diet for type 2 diabetic patients), as depicted in Supplementary Figure S2.

## 5 Discussion

This review included a total of 12 dietary intervention trials. In the comprehensive meta-analysis, alterations in glucose metabolism and BMI within the experimental group (adhering to nutrient intake adjusted according to the recommended diet for individuals with T2D) did not demonstrate statistical significance when compared to the control group (following the recommended diet for T2D patients). However, the experimental group exhibited noteworthy reductions in both total cholesterol and LDL-C levels. In subgroup analyses, it was observed that FBG, HbA1c, and total cholesterol were significantly lower following interventions involving high dietary fiber diets, such as the Ma-PI^2^ diet, high-fiber diet, and Mediterranean diet, when contrasted with pre-intervention levels. Furthermore, HbA1c exhibited a significant decrease after high-fat, low-carbohydrate diet interventions, as seen in the ketogenic diet and almond-based low-carbohydrate diet. Notably, the low-fat and low-carbohydrate diet, i.e., a low-energy diet, significantly enhanced glucose metabolism levels (FBG and HOMA-IR), as well as general and central obesity, as measured by BMI and waist circumference, in overweight and obese T2D patients. Furthermore, a personalized diet tailored to the individual's gut microbiota, immune system, and biochemical parameters demonstrated superior efficacy in glycemic control among T2D patients, leading to a more diverse population of beneficial gut bacteria than the specific diets previously mentioned.

The present review appears to provide further evidence of an earlier study by Houghton et al. (27), a systematic review evaluating the effectiveness of dietary interventions on the gut microbiota and glycemic control in adults with type 2 diabetes mellitus. Houghton et al. found a significant reduction in HbA1c and no significant changes in FBG or HOMA-IR in patients after dietary intervention. In terms of the gut microbiota, there were significant changes in diversity matrices (α and β) and the Firmicutes: Bacteroidetes ratios, but no significant changes in the relative abundance of Bifidobacterium spp. However, the present review builds on that study by updating the intervention studies of the last few years and analyzing subgroups according to nutritional characteristics, culminating in further results on the gut microbiota.

In comparison to the healthy population, patients with T2DM present a diminished abundance and diversity of gut microbiota, specifically lacking in butyrate-producing bacteria (e.g., *Ruminococcus*, Subdoligranulum, *Eubacterium, Faecalibacterium prausnitzii*, and *Roseburia*) and bacteria inversely associated with inflammation (*Bacteroides, Prevotella, Akkermansia*, and *Bifidobacterium*) (28). Noteworthy, changes in the gut microbiota appear to precede alterations in the standard biomarker of type 2 diabetes, HbA1c (17). The consumption of a Western-style diet, characterized by elevated levels of refined sugars, carbohydrates, saturated fatty acids, and animal proteins, coupled with a low dietary fiber intake, correlates with inflammation, metabolic disease, and T2DM (29). Remarkable traits of the Western-style diet-associated gut microbiota include an upsurge in protein-metabolizing bacteria (e.g., Bacillus and Aspergillus), saturated fat-metabolizing bacteria (e.g., Bacillus spp.), and a substantial reduction in fiber-degrading bacteria (e.g., *Faecalibacterium* and *Lachnospira*) (30, 31). Following the consumption of red meat, the gut microbiota ferments its constituent choline, carnitine, betaine, and lecithin, resulting in the synthesis of trimethylamine (TMA). Subsequently, the liver further metabolizes TMA to trimethylamine-N-oxide (TMAO) (32). A study demonstrated that TMAO impairs glucose tolerance, elevates HOMA-IR and fasting insulin levels in mice fed a high-fat diet, inducing adipose tissue inflammation and blocking insulin signaling (33). Furthermore, a case-control study revealed a positive correlation between increased plasma TMAO levels and heightened risk of T2DM (34). Therefore, adopting a rational and effective dietary pattern stands as a powerful means to augment gut microbiota diversity, while balancing its composition and metabolism in patients with T2DM.

Dietary interventions examined in this review exhibited a significant impact on the diversity of the gut microbiota. Various interventions, such as low-energy, low-carbohydrate, low-fat, and high-fiber Ma-Pi 2 diets, were found to notably increase alpha diversity. Moreover, the Mediterranean diet and ketogenic diet demonstrated a significant increase in beta diversity of the gut microbiota. Notably, several studies indicated a strong association between bacterial fluctuations resulting from dietary interventions and improved metabolic pathways, including the degradation pathways of limonene and ethylbenzene, glycosaminoglycan, and unsaturated fatty acid biosynthesis (12, 16, 18).

Importantly, the majority of dietary interventions significantly modify the composition of the gut microbiota, with some of the altered flora closely linked to human metabolic function. Specifically, high-fiber Ma-Pi 2 diets, high dietary fiber diet, high-fiber low-energy diet, and Mediterranean diet, all belonging to high dietary fiber categories, upregulated the relative abundance of *Bacteroides, Faecalibacterium prausnitzii, Akkermansia*, and *Bifidobacterium*. *Faecalibacterium prausnitzii*, a major SCFA (such as acetic and butyric acid) producer in the human intestine, notably enhances insulin sensitivity and ameliorates T2DM (35). Additionally, numerous *Firmicutes* members, including *Lachnospira, Pseudobutyrivibrio, Clostridium leptum, Roseburia*, and *Faecalibacterium*, possess robust SCFA-producing capabilities (36). SCFA stimulates insulin secretion from pancreatic β-cells by stimulating the release of glucagon-like peptide (GLP-1) and casein (PPY) from intestinal L-cells and reduces inflammation levels by inhibiting indole- and hydrogen sulfide-producing bacteria (13, 37, 38).

However, the majority of bacterial strains commonly found in today's probiotic supplements do not possess the ability to produce butyrate. This limitation stems from the fact that most butyrate-producing bacteria are highly anaerobic and perish rapidly upon exposure to oxygen. In contrast, direct administration of butyrate can be absorbed by the stomach (36). While it is not possible to directly supplement butyrate-producing bacteria or butyrate itself, it is feasible to nourish butyric acid bacteria within the gut through dietary intake. This indirect approach facilitates an increase in the abundance of butyrate-producing bacteria and stimulates their substantial production of short-chain fatty acids (SCFAs). The consumption of dietary fiber represents the optimal means of augmenting SCFA-producing bacteria. Dietary fiber predominantly encompasses cellulose, resistant starch, pectin, inulin, and oligosaccharides, with whole grains, legumes, nuts, vegetables, and fruits constituting major food sources.

Secondly, *Akkermansia muciniphila* (*A. Muciniphila*) has been the subject of increasing research due to its diminished abundance in patients with diabetes, cardiovascular disease, inflammatory bowel disease, and neurological disorders (39, 40). *A. Muciniphila* may stimulate increased levels of glucagon-like peptide-1 (GLP-1) through protein P9 on the outer membrane, thereby promoting insulin secretion from pancreatic β-cells and suppressing appetite, ultimately improving T2DM and obesity (41). The most effective approach to enhancing *A. Muciniphila* abundance in the gut involves consuming foods rich in polyphenols and fish oil, alongside a dietary fiber intake (42). Notably, polyphenols act as antioxidants, combating oxidative stress and chronic inflammation, while also improving insulin resistance. Foods such as flax seeds, rye bread, walnuts, cranberries, blueberries, and green tea are abundant sources of dietary polyphenols (43). Additionally, fish such as sardines and salmon not only provide fish oil (DHA) but also serve as excellent sources of high-quality protein (44). Our study revealed that a high dietary, low-energy regimen enriched with polyphenols and plant proteins, as designed by Medina-Vera et al. (15), resulted in a 125% increase in *A. Muciniphila* abundance, a 65% decrease in its endotoxin (LSP) concentration, and a significant enhancement of plasma antioxidant activity. Furthermore, *Bifidobacterium* is extensively utilized in fermented dairy products as one of the most prevalent probiotics for promoting healthy intestinal function in humans (45), while *Bacteroides fragilis* exhibits a robust capacity for the extensive breakdown of dietary fiber polysaccharides and host glycans (15).

We observed a decline in *Prevotella* abundance in two high dietary fiber-based diets (14, 15), with a 13% reduction in *Prevotella copri* specifically in the high dietary fiber low-energy diet. Similarly, an animal study (46) and a population intervention trial (47) demonstrated an association between *Prevotella copri* and insulin resistance. Conversely, numerous clinical trials have consistently reported an elevation in *Prevotella* abundance following high dietary fiber interventions (48–51). Moreover, one study revealed the potential benefits of *Prevotella copri* in host metabolism, suggesting its utility as an indicator of postprandial glucose metabolism (52). Nevertheless, the precise effects of *Prevotella* on human health and its underlying mechanisms remain unclear. Potential factors contributing to this discrepancy include inter-individual variability in species and strain-level composition of *Prevotella* within the gut and variations in dietary patterns (53). Thus, the prevailing explanation is that higher diversity of *Prevotella spp*. species corresponds to a greater fermentative capacity, yielding greater benefits for human health (53).

In addition to dietary fiber and polyphenols, the gut microbiota play a vital role in lipid metabolism, encompassing lipid conversion, synthesis, breakdown of dietary lipids, and generation of host-regulated secondary metabolites (54). The Mediterranean diet, rich in n-3 polyunsaturated fatty acids (n-3 PUFA) abundant in fish, is strongly linked to improved T2DM outcomes (55). n-3 PUFA exhibit anti-inflammatory properties by reducing the *Lachnospira*ceae/*Firmicutes* ratio and enhancing *Lachnospira*ceae, thereby interacting with the gut microbiota to suppress inflammation. These effects are particularly attributed to the reduction of lipopolysaccharide (LPS)-producing bacteria and the increase in short-chain fatty acid (SCFA)-producing bacteria (56, 57). However, the three studies (17, 18, 20) included in this paper, which explored dietary interventions enriched in n-3 PUFA (Mediterranean diet and sardine diet), did not consistently demonstrate alterations in gut microbiota composition, suggesting incomplete correlation with reported results of gut microbiota changes.

Regarding the reduced branched-chain amino acid (BCAA) diet, this paper only includes one study (21). The findings indicate that compared to a full BCAA diet, reduced BCAA intake leads to increased postprandial insulin sensitivity, improved gut microbiota composition, and enhanced white adipose tissue metabolism. BCAA, an essential amino acid synthesized by the gut microbiota, has emerged as a biomarker for insulin resistance (58). However, a two-way Mendelian randomization study demonstrated a causal association between insulin resistance and higher BCAA levels, whereas higher BCAA levels were not causally associated with insulin resistance (59).

**Figure 4:**
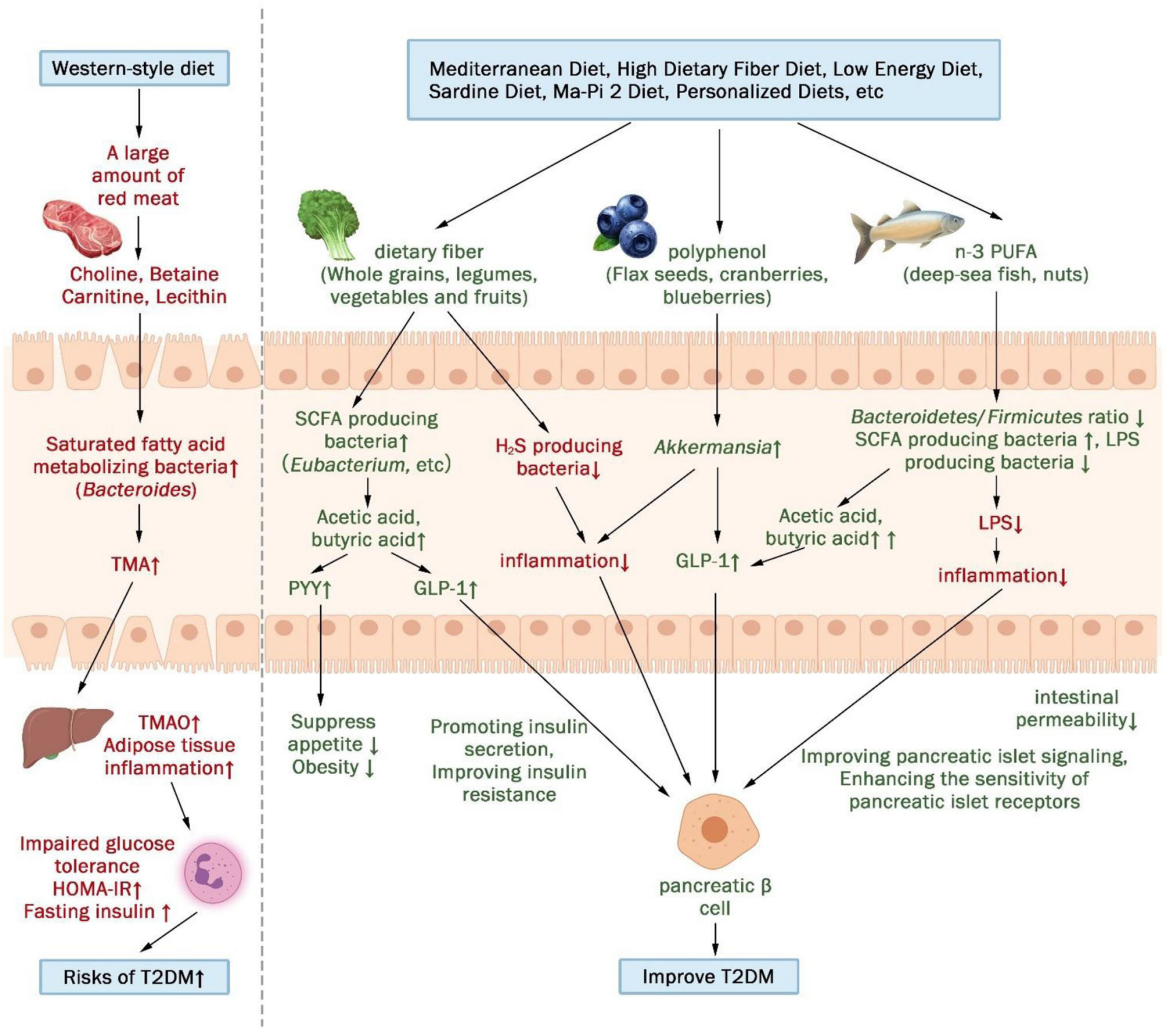
The gut microbiota and its metabolites may act as a mediator between diet and metabolism in patients with T2DM. (↑indicates increase, ↓indicates decrease).

In summary, the gut microbiota and their metabolites serve as potential mediators between diet and T2DM metabolism (Figure 4). This pivotal connection was also demonstrated in the context of a mediation analysis pertaining to personalized dietary interventions (23), where alterations in the gut microbiome composition elucidated 12.25% of the variations observed in serum metabolites. A Western diet, positively associated with T2DM, promotes impaired glucose tolerance by fostering the growth of saturated fatty acid-metabolizing bacteria and triggering the secretion of trimethylamine N-oxide (TMAO), ultimately elevating the risk of T2DM. Conversely, diets negatively associated with T2DM promotes insulin secretion and improves insulin resistance by increasing SCFA-producing bacteria and decreasing H2S- and LPS-producing bacteria.

The human gut microbiota is intricately associated with dietary patterns and influenced by confounding factors such as age, race, gender, geography, and socioeconomic variables (60). Furthermore, it can be substantially impacted by different disease stages and treatment medications (60, 61). A Meta-analysis that included 27 studies (62) showed that age is an important factor influencing the diversity, composition and functional characteristics of the gut microbiota, and in particular, beta diversity varies significantly across developmental stages.

The growing recognition of the gut microbiome's predictive capacity for human health necessitates its incorporation as a foundation for personalized health management indicators (63). Moreover, diet-microbiota interactions underpin the advancement of precision nutrition, where the gut microbiota composition emerges as a critical factor in determining responses to dietary interventions (64). Therefore, by integrating gut microbiomics and metabolomics with robust data analysis, machine learning algorithms can aid in devising personalized diets that offer more effective guidance for the prevention and management of T2DM through nutritional interventions (65). This paper includes a study featuring a personalized diet intervention designed by Meleshko et al. (22), which tailored the diet based on the individuals' gut microbiota status, immune responses, and biochemical parameters among T2DM patients. The results showed significant improvements in blood glucose levels, lipid profiles, and inflammatory markers, along with notable reductions in metabolism-related detrimental bacteria (e.g., *Enterococcus faecalis, Escherichia coli, lac*+, and *Candida spp*.). Shoer et al. (23) found that personalized diets had greater beneficial effects on glycemic control, gut microbiota and metabolites than the Mediterranean diet.

Nevertheless, there are several current challenges and issues associated with employing precision nutrition for disease prevention and management. These include the high cost of histological techniques, the complexity of study design methods, the analysis and interpretation of high-dimensional data, and result reproducibility (66, 67). In the face of these challenges, future research efforts should prioritize rigorous and rational study designs, cost reduction of histological technique analyses, and the development of algorithms capable of handling high-dimensional big data from diverse sources (67).

## 6 Advantages and limitations

To our knowledge, this is the inaugural meta-analysis and systematic review designed to categorize dietary interventions based on their nutritional attributes, aiming to evaluate their efficacy in ameliorating the gut microbiota and metabolic profiles of individuals with T2DM. This approach holds broader instructive value for individuals in their daily dietary choices. Additionally, we elucidate the intermediary role of the gut microbiota and its metabolites in bridging dietary patterns and T2DM. In this context, as different diets influence the gut microbiota, they subsequently leverage various pathways to enhance the metabolic status of T2DM patients. We should note that our scope of interventions excludes isolated nutritional supplements and probiotic interventions. The inclusion of probiotics, considered beneficial bacteria, could directly interfere with the impact of dietary interventions on the gut microbiota. Moreover, nutritional supplements are not commonly integrated into individuals' daily dietary habits.

However, it is imperative to acknowledge that our meta-analysis incorporated a substantial degree of heterogeneity, primarily attributed to the variance in the methodologies employed for dietary interventions. Additionally, patient age, variations in disease progression, intervention duration combined with medication usage, and the limited sample sizes within the studies could potentially obscure the genuine alterations in gut microbiota and metabolic markers. It is pertinent to mention that the relatively limited number of meta-analyses focusing on gut microbiota is due to the scarcity of specific gut microbiota-related variables accessible for complete experimental and control groups. In certain instances, studies solely compared changes between the intervention and baseline levels, further contributing to this scarcity.

## 7 Conclusion and prospect

Regarding specific dietary patterns, a high dietary fiber regimen led to a noteworthy reduction in FBG, HbA1c, and total cholesterol levels while augmenting the prevalence of short-chain fatty acid-producing bacteria. The high-fat and low-carbohydrate diet was particularly effective in lowering HbA1c levels, and a low-fat, low-carbohydrate diet exhibited significant reductions in FBG, HOMA-IR, BMI, and waist circumference. Notably, individualized dietary strategies demonstrated superior efficacy in blood glucose management among T2D patients when compared to predefined dietary patterns. This approach also fostered a greater diversity of beneficial gut bacteria.

In summary, diverse dietary interventions can enhance metabolic profiles by positively influencing the gut microbiota, consequently leading to improvements in metabolic parameters among individuals with T2D.

Consequently, future clinical investigations are warranted to comprehensively explore the effects of dietary modalities on the gut microbiota of T2DM patients, as well as establish connections between alterations in the gut microbiota and changes in T2DM-associated biochemical markers. In forthcoming studies, it is imperative to consider appropriate sample sizes, extend intervention durations, and continuously monitor the dynamics of the gut microbiota and its metabolites. Additionally, it is essential to move beyond bacterial classification and perform functional group analyses of functionally similar microorganisms when studying the gut microbiota. Regarding dietary interventions, apart from commonly studied patterns such as the Mediterranean diet, high-fiber diets, and low-energy diets, future investigations should embrace personalized and tailored approaches based on individual variations in gut microbiota characteristics, immune and biochemical indicators, disease stage, and drug response diversity.

## Data availability statement

The original contributions presented in the study are included in the article/Supplementary material, further inquiries can be directed to the corresponding author.

## Author contributions

Conceptualization: XX and HX. Methodology: XX, FZ, JR, HZ, CJ, MW, and YJ. Validation: XX, FZ, and JR. Formal analysis and writing—original draft preparation: XX. Writing—review and editing: XX, HX, FZ, JR, HZ, CJ, MW, and YJ. All authors have read and agreed to the published version of the manuscript.

## References

[1] Chatterjee S Khunti K Davies MJ. Type 2 diabetes. Lancet. (2017) 389:2239–51. 10.1016/S0140-6736(17)30058-2 28190580

[2] Gurung M Li Z You H Rodrigues R Jump DB Morgun A . Role of gut microbiota in type 2 diabetes pathophysiology. EBioMedicine. (2020) 51:102590. 10.1016/j.ebiom.2019.11.051 31901868 PMC6948163

[3] Yang G Wei J Liu P Zhang Q Tian Y Hou G . Role of the gut microbiota in type 2 diabetes and related diseases. Metabolism. (2021) 117:154712. 10.1016/j.metabol.2021.154712 33497712

[4] Sohail MU Althani A Anwar H Rizzi R Marei HE. Role of the gastrointestinal tract microbiome in the pathophysiology of diabetes mellitus. J Diabetes Res. (2017) 2017:1–9. 10.1155/2017/9631435 29082264 PMC5634576

[5] Koren O Goodrich JK Cullender TC Spor A Laitinen K Kling Bäckhed H . Host Remodeling of the Gut Microbiome and Metabolic Changes during Pregnancy. Cell. (2012) 150:470–80. 10.1016/j.cell.2012.07.008 22863002 PMC3505857

[6] Brown JM Hazen SL. The gut microbial endocrine organ: bacterially derived signals driving cardiometabolic diseases. Annu Rev Med. (2015) 66:343–59. 10.1146/annurev-med-060513-093205 25587655 PMC4456003

[7] Zhu T Goodarzi MO. Metabolites linking the gut microbiome with risk for type 2 diabetes. Curr Nutr Rep. (2020) 9:83–93. 10.1007/s13668-020-00307-3 32157661 PMC7282969

[8] Ganesan K Chung SK Vanamala J Xu B. Causal relationship between diet-induced gut microbiota changes and diabetes: a novel strategy to transplant faecalibacterium prausnitzii in preventing diabetes. IJMS. (2018) 19:3720. 10.3390/ijms19123720 30467295 PMC6320976

[9] Mozaffarian D. Dietary D and policy priorities for cardiovascular disease, diabetes, and obesity: a comprehensive review. Circulation. (2016) 133:187–225. 10.1161/CIRCULATIONAHA.115.018585 26746178 PMC4814348

[10] Hodge A Bassett J. What can we learn from dietary pattern analysis? Public Health Nutr. (2016) 19:191–4. 10.1017/S1368980015003730 26784585 PMC10273249

[11] Page MJ McKenzie JE Bossuyt PM Boutron I Hoffmann TC Mulrow CD . The PRISMA 2020 statement: an updated guideline for reporting systematic reviews. BMJ. (2021) 2021:n71. 10.1136/bmj.n71 PMC8005924 33782057

[12] Candela M Biagi E Soverini M Consolandi C Quercia S Severgnini M . Modulation of gut microbiota dysbioses in type 2 diabetic patients by macrobiotic Ma-Pi 2 diet. Br J Nutr. (2016) 116:80–93. 10.1017/S0007114516001045 27151248 PMC4894062

[13] Zhao L Zhang F Ding X Wu G Lam YY Wang X . Gut bacteria selectively promoted by dietary fibers alleviate type 2 diabetes. Science. (2018) 359:1151–6. 10.1126/science.aao5774 29590046

[14] Chen L Liu B Ren L Du H Fei C Qian C . High-fiber diet ameliorates gut microbiota, serum metabolism and emotional mood in type 2 diabetes patients. Front Cell Infect Microbiol. (2023) 13:1069954. 10.3389/fcimb.2023.1069954 36794003 PMC9922700

[15] Medina-Vera I Sanchez-Tapia M Noriega-López L Granados-Portillo O Guevara-Cruz M Flores-López A . A dietary intervention with functional foods reduces metabolic endotoxaemia and attenuates biochemical abnormalities by modifying faecal microbiota in people with type 2 diabetes. Diabetes Metab. (2019) 45:122–31. 10.1016/j.diabet.2018.09.004 30266575

[16] Jian C Silvestre MP Middleton D Korpela K Jalo E Broderick D . Gut microbiota predicts body fat change following a low-energy diet: a PREVIEW intervention study. Genome Med. (2022) 14:54. 10.1186/s13073-022-01053-7 35599315 PMC9125896

[17] Ismael S Silvestre MP Vasques M Araújo JR Morais J Duarte MI . A pilot study on the metabolic impact of mediterranean diet in type 2 diabetes: is gut microbiota the key? Nutrients. (2021) 13:1228. 10.3390/nu13041228 33917736 PMC8068165

[18] Deledda A Palmas V Heidrich V Fosci M Lombardo M Cambarau G . Dynamics of gut microbiota and clinical variables after ketogenic and mediterranean diets in drug-naïve patients with type 2 diabetes mellitus and obesity. Metabolites. (2022) 12:1092. 10.3390/metabo12111092 36355175 PMC9693465

[19] Ren M Zhang H Qi J Hu A Jiang Q Hou Y . An almond-based low carbohydrate diet improves depression and glycometabolism in patients with type 2 diabetes through modulating gut microbiota and GLP-1: a randomized controlled trial. Nutrients. (2020) 12:3036. 10.3390/nu12103036 33022991 PMC7601479

[20] Balfegó M Canivell S Hanzu FA Sala-Vila A Martínez-Medina M Murillo S . Effects of sardine-enriched diet on metabolic control, inflammation and gut microbiota in drug-naïve patients with type 2 diabetes: a pilot randomized trial. Lipids Health Dis. (2016) 15:78. 10.1186/s12944-016-0245-0 27090218 PMC4836051

[21] Karusheva Y Koessler T Strassburger K Markgraf D Mastrototaro L Jelenik T . Short-term dietary reduction of branched-chain amino acids reduces meal-induced insulin secretion and modifies microbiome composition in type 2 diabetes: a randomized controlled crossover trial. Am J Clin Nutr. (2019) 110:1098–107. 10.1093/ajcn/nqz191 31667519 PMC6821637

[22] Meleshko T Rukavchuk R Levchuk O Boyko N. Personalized nutrition for microbiota correction and metabolism restore in type 2 diabetes mellitus patients. In:Donelli G, editor. Advances in Microbiology, Infectious Diseases and Public Health. Cham: Advances in Experimental Medicine and Biology; Springer International Publishing. (2021). 10.1007/5584_2021_621 33634376

[23] Shoer S Shilo S Godneva A Ben-Yacov O Rein M Wolf BC . Impact of dietary interventions on pre-diabetic oral and gut microbiome, metabolites and cytokines. Nat Commun. (2023) 14:5384. 10.1038/s41467-023-41042-x 37666816 PMC10477304

[24] Wan X Wang W Liu J Tong T. Estimating the sample mean and standard deviation from the sample size, median, range and/or interquartile range. BMC Med Res Methodol. (2014) 14:135. 10.1186/1471-2288-14-135 25524443 PMC4383202

[25] Luo D Wan X Liu J Tong T. Optimally estimating the sample mean from the sample size, median, mid-range, and/or mid-quartile range. Stat Methods Med Res. (2018) 27:1785–805. 10.1177/0962280216669183 27683581

[26] Higgins J Green S. Cochrane Handbook for Systematic Reviews of Interventions Version 5.1.0 [Updated March 2011]. London: The Cochrane Collaboration. (2011).

[27] Houghton D Hardy T Stewart C Errington L Day CP Trenell MI . Systematic review assessing the effectiveness of dietary intervention on gut microbiota in adults with type 2 diabetes. Diabetologia. (2018) 61:1700–11. 10.1007/s00125-018-4632-0 29754286 PMC6061157

[28] Cunningham AL Stephens JW Harris DA. Gut Microbiota Influence in Type 2 Diabetes Mellitus (T2DM). Gut Pathog. (2021) 13:50. 10.1186/s13099-021-00446-0 34362432 PMC8343927

[29] Janssen JA. Hyperinsulinemia MJL, and its pivotal role in aging, obesity, type 2 diabetes, cardiovascular disease and cancer. IJMS. (2021) 22:7797. 10.3390/ijms22157797 34360563 PMC8345990

[30] Hills R Pontefract B Mishcon H Black C Sutton S Theberge C. Gut microbiome: profound implications for diet and disease. Nutrients. (2019) 11:1613. 10.3390/nu11071613 31315227 PMC6682904

[31] Malesza IJ Malesza M Walkowiak J Mussin N Walkowiak D Aringazina R . High-fat western-style diet, systemic inflammation, and gut microbiota: a narrative review. Cells. (2021) 10:3164. 10.3390/cells10113164 34831387 PMC8619527

[32] Bennet B Vallim T Wang Z Shih D Meng Y Gregory J . Trimethylamine-N-oxide, a metabolite associated with atherosclerosis, exhibits complex genetic and dietary regulation. Cell Metab. (2013) 17:49–60. 10.1016/j.cmet.2012.12.011 23312283 PMC3771112

[33] Gao X Liu X Xu J Xue C Xue Y Wang Y. Dietary trimethylamine n-oxide exacerbates impaired glucose tolerance in mice fed a high fat diet. J Biosci Bioeng. (2014) 118:476–81. 10.1016/j.jbiosc.2014.03.001 24721123

[34] Association between Microbiota-Dependent Metabolite Trimethylamine-N-Oxide Type 2 Diabetes – ScienceDirect. Available online at: https://www.sciencedirect.com/science/article/pii/S0002916522026077?via%3Dihub (accessed 2 April, 2023).

[35] Mukherjee A Lordan C Ross RP Cotter PD. Gut microbes from the phylogenetically diverse genus eubacterium and their various contributions to gut health. Gut Microbes. (2020) 12:1802866. 10.1080/19490976.2020.1802866 32835590 PMC7524325

[36] Arora T Tremaroli V. Therapeutic potential of butyrate for treatment of type 2 diabetes. Front Endocrinol. (2021) 12:761834. 10.3389/fendo.2021.761834 34737725 PMC8560891

[37] Udayappan S Manneras-Holm L Chaplin-Scott A Belzer C Herrema H Dallinga-Thie GM . Oral treatment with eubacterium hallii improves insulin sensitivity in db/db mice. NPJ Biofilms Microbiomes. (2016) 2:16009. 10.1038/npjbiofilms.2016.9 28721246 PMC5515273

[38] Li K-K Tian P-J Wang S-D Lei P Qu L Huang J-P . Targeting gut microbiota: lactobacillus alleviated type 2 diabetes via inhibiting lps secretion and activating GPR43 pathway. J Funct Foods. (2017) 38:561–70. 10.1016/j.jff.2017.09.049

[39] Depommier C Everard A Druart C Plovier H Van Hul M Vieira-Silva S . Supplementation with akkermansia muciniphila in overweight and obese human volunteers: a proof-of-concept exploratory study. Nat Med. (2019) 25:1096–103. 10.1038/s41591-019-0495-2 31263284 PMC6699990

[40] Hasani A Ebrahimzadeh S Hemmati F Khabbaz A Hasani A Gholizadeh P. The role of akkermansia muciniphila in obesity, diabetes and atherosclerosis. J Med Microbiol. (2021) 70:1435. 10.1099/jmm.0.001435 34623232

[41] Cani PD Depommier C Derrien M Everard A de Vos WM. Akkermansia muciniphila: paradigm for next-generation beneficial microorganisms. Nat Rev Gastroenterol Hepatol. (2022) 19:625–37. 10.1038/s41575-022-00631-9 35641786

[42] Zhou K. Strategies to promote abundance of akkermansia muciniphila, an emerging probiotics in the gut, evidence from dietary intervention studies. J Funct Foods. (2017) 33:194–201. 10.1016/j.jff.2017.03.045 30416539 PMC6223323

[43] Wan MLY Co VA El-Nezami H. Dietary polyphenol impact on gut health and microbiota. Crit Rev Food Sci Nutr. (2021) 61:690–711. 10.1080/10408398.2020.1744512 32208932

[44] Ghasemi Fard S Wang F Sinclair AJ Elliott G Turchini GM. How Does High DHA Fish Oil Affect Health? A systematic review of evidence critical reviews in food science and nutrition. Crit Rev Food Sci Nutr. (2019) 59:1684–727. 10.1080/10408398.2018.1425978 29494205

[45] Hidalgo-Cantabrana C Delgado S Ruiz L Ruas-Madiedo P Sánchez B. Margolles, bifidobacteria a, and their health-promoting effects. Microbiol Spectr. (2017) 5:21. 10.1128/microbiolspec.BAD-0010-2016 28643627 PMC11687494

[46] Pedersen HK Gudmundsdottir V Nielsen HB Hyotylainen T Nielsen T Jensen BAH . Human gut microbes impact host serum metabolome and insulin sensitivity. Nature. (2016) 535:376–81. 10.1038/nature18646 27409811

[47] Meslier V Laiola M Roager HM De Filippis F Roume H Quinquis B . Mediterranean diet intervention in overweight and obese subjects lowers plasma cholesterol and causes changes in the gut microbiome and metabolome independently of energy intake. Gut. (2020) 69:1258–68. 10.1136/gutjnl-2019-320438 32075887 PMC7306983

[48] Haro C García-Carpintero S Rangel-Zúñiga OA Alcalá-Díaz JF Landa BB Clemente JC . Consumption of two healthy dietary patterns restored microbiota dysbiosis in obese patients with metabolic dysfunction. Mol Nutr Food Res. (2017) 61:1700300. 10.1002/mnfr.201700300 28940737

[49] Marungruang N Tovar J Björck I Hållenius FF. Improvement in cardiometabolic risk markers following a multifunctional diet is associated with gut microbial taxa in healthy overweight and obese subjects. Eur J Nutr. (2018) 57:2927–36. 10.1007/s00394-017-1563-3 29098426 PMC6267413

[50] Ghosh TS Rampelli S Jeffery IB Santoro A Neto M Capri M . Mediterranean diet intervention alters the gut microbiome in older people reducing frailty and improving health status: the nu-age 1-year dietary intervention across five european countries. Gut. (2020) 69:1218–28. 10.1136/gutjnl-2019-319654 32066625 PMC7306987

[51] Roager HM Vogt JK Kristensen M Hansen LBS Ibrügger S Mærkedahl RB . Whole grain-rich diet reduces body weight and systemic low-grade inflammation without inducing major changes of the gut microbiome: a randomised cross-over trial. Gut. (2019) 68:83–93. 10.1136/gutjnl-2017-314786 29097438 PMC6839833

[52] Asnicar F Berry SE Valdes AM Nguyen LH Piccinno G Drew DA . Microbiome connections with host metabolism and habitual diet from 1,098 deeply phenotyped individuals. Nat Med. (2021) 27:321–32. 10.1038/s41591-020-01183-8 33432175 PMC8353542

[53] Tett A Pasolli E Masetti G Ercolini D Segata N. Prevotella diversity niches and interactions with the human host. Nat Rev Microbiol. (2021) 19:585–99. 10.1038/s41579-021-00559-y 34050328 PMC11290707

[54] Brown EM Clardy J Xavier RJ. Gut microbiome lipid metabolism and its impact on host physiology. Cell Host Microbe. (2023) 31:173–86. 10.1016/j.chom.2023.01.009 36758518 PMC10124142

[55] Oliver E McGillicuddy F Phillips C Toomey S Roche HM. The role of inflammation and macrophage accumulation in the development of obesity-induced type 2 diabetes mellitus and the possible therapeutic effects of long-chain n-3 PUFA. Proc Nutr Soc. (2010) 69:232–43. 10.1017/S0029665110000042 20158940

[56] Watson H Mitra S Croden FC Taylor M Wood HM Perry SL . A Randomised trial of the effect of omega-3 polyunsaturated fatty acid supplements on the human intestinal microbiota. Gut. (2018) 67:1974–83. 10.1136/gutjnl-2017-314968 28951525

[57] Costantini L Molinari R Farinon B Merendino N. Impact of omega-3 fatty acids on the gut microbiota. IJMS. (2017) 18:2645. 10.3390/ijms18122645 29215589 PMC5751248

[58] Ruiz-Canela M Guasch-Ferré M Toledo E Clish CB Razquin C Liang L . Plasma branched chain/aromatic amino acids, enriched mediterranean diet and risk of type 2 diabetes: case-cohort study within the PREDIMED trial. Diabetologia. (2018) 61:1560–71. 10.1007/s00125-018-4611-5 29663011 PMC5988977

[59] Mahendran Y Jonsson A Have CT Allin KH Witte DR Jørgensen ME . Genetic Evidence of a Causal Effect of Insulin Resistance on Branched-Chain Amino Acid Levels. Diabetologia. (2017) 60:873–8. 10.1007/s00125-017-4222-6 28184960

[60] Zhang J Guo Z Xue Z Sun Z Zhang M Wang L . A phylo-functional core of gut microbiota in healthy young chinese cohorts across lifestyles, geography and ethnicities. ISME J. (2015) 9:1979–90. 10.1038/ismej.2015.11 25647347 PMC4542028

[61] Whang A Nagpal R Yadav H. Bi-directional drug-microbiome interactions of anti-diabetics. EBioMedicine. (2019) 39:591–602. 10.1016/j.ebiom.2018.11.046 30553752 PMC6354569

[62] Badal VD Vaccariello ED Murray ER Yu KE Knight R Jeste DV . The gut microbiome, aging, and longevity: a systematic review. Nutrients. (2020) 12:3759. 10.3390/nu12123759 33297486 PMC7762384

[63] Larsen PE Dai Y. Metabolome of human gut microbiome is predictive of host dysbiosis. GigaSci. (2015) 4:42. 10.1186/s13742-015-0084-3 26380076 PMC4570295

[64] Biesiekierski JR Jalanka J Staudacher HM. Can gut microbiota composition predict response to dietary treatments? Nutrients. (2019) 11:1134. 10.3390/nu11051134 31121812 PMC6566829

[65] de Toro-Martín J Arsenault B Després J-P Vohl M-C. Precision nutrition: a review of personalized nutritional approaches for the prevention and management of metabolic syndrome. Nutrients. (2017) 9:913. 10.3390/nu9080913 28829397 PMC5579706

[66] Merino J. Precision nutrition in diabetes: when population-based dietary advice gets personal. Diabetologia. (2022) 65:1839–48. 10.1007/s00125-022-05721-6 35593923

[67] Wang DD Hu FB. Precision nutrition for prevention and management of type 2 diabetes. Lancet Diab Endocrinol. (2018) 6:416–26. 10.1016/S2213-8587(18)30037-8 29433995

