# Corrigendum: Dietary intervention improves metabolic levels in patients with type 2 diabetes through the gut microbiota: a systematic review and meta-analysis

**DOI:** 10.3389/fnut.2024.1414687

**Published:** 2024-04-29

**Authors:** Xiaoyu Xu, Fan Zhang, Jiajia Ren, Haimeng Zhang, Cuiqi Jing, Muhong Wei, Yuhong Jiang, Hong Xie

**Affiliations:** ^1^School of Public Health, Bengbu Medical University, Bengbu, China; ^2^Department of Epidemiology and Health Statistics, School of Public Health, Bengbu Medical University, Bengbu, China; ^3^Department of Nutrition and Food Hygiene, School of Public Health, Bengbu Medical University, Bengbu, China

**Keywords:** type 2 diabetes, dietary intervention, gut microbiota, short-chain fatty acids, systematic review

In the published article, there was an error in Table 1 as published. There is a nationality error in the first column and last row of Table 1. The mistake read as “Shoer et al. (23) Palestine”. This should have been written as “Shoer et al. (23) Israel”. The corrected Table 1 appears below.

**Table 1:** Description and characteristics of the included studies.

**References/** **Country**	**Dietary patterns and groupings**	**Nutritional characteristics of the intervention group**	**Study Design**	**Subject** **characteristics**	**Main microbiota results**	**Main clinical outcomes**
Candela et al. (12) Italy	Experimental group: fiber-rich longevity Ma-Pi 2 diet (*n* = 28) Control group: Italian Professional Association for T2D Therapy recommended diet CTR (*n* = 28) Healthy group: normal diet (*n* = 13)	High dietary fiber	Open randomized controlled trial, 21 days	T2DM, *n* = 56, BMI of 27–45 kg/m^2^, 55–70 years old; Healthy group: normal weight, 21-40 years old.	Both dietary interventions demonstrated effectiveness in alleviating gut microbiota dysbiosis and promoting the restoration of bacteria that produce short-chain fatty acids (SCFAs) in individuals with T2DM.	The reduction in HOMA-IR, total cholesterol and LDL/HDL ratio was significantly higher in the Ma-Pi 2 diet group than in the CTR group. the Ma-Pi 2 diet significantly reduced TNF-α, plasma CRP and IL-6 levels, while only TNF-α was significantly reduced in the CTR group.
Zhao et al. (13) China	Experimental group: high dietary fiber diet (*n* = 27) Control group: 2013 version of the Chinese Diabetes Association dietary guidelines for patients to manage their diet (*n* = 16)	High dietary fiber	Randomized controlled trial, 84 days	T2DM, *n* = 43, acarbose as a treatment drug	The high dietary fiber intervention increased 15 strains of acetate and butyric acid-producing bacteria, inhibited perindole- and hydrogen sulfide-producing bacteria, and promoted GLP-1 and PYY secretion to improve blood glucose, the abundance and diversity of which correlated significantly with clinical outcomes.	Indicators such as HbA1c improved faster and better in the experimental group than in the control group, and this clinical effect could be reproduced in mice by colony transplantation.
Chen et al. (14) China	Experimental group: high dietary fiber diet (*n* = 9) Control group: 2013 version of the Chinese Diabetes Association dietary guidelines for patients to manage their diet (*n* = 8)	High dietary fiber	Randomized controlled trial, 4 weeks	T2DM, *n* = 17, acarbose as a therapeutic agent	The ratio of *Firmicutes* to *Bacteroidota* was significantly lower in the treatment group, and the number of *Proteus* was reduced; the proportion of beneficial microorganisms of several genera increased, and the relative abundance of all other opportunistic pathogens decreased.	Glucose homeostasis, glucose homeostasis and systemic inflammation levels were significantly improved in the treatment group compared to the control group.
Medina-Vera et al. (15) Mexico	Experimental group: Functional food diet (*n* = 81) (T2DM) Control group: normal diet (healthy population)	High dietary fiber, low carb, high unsaturated fat	Randomized controlled trial, 12 weeks	T2DM, *n* = 81, 30-60 years old, and BMI of 18.5-24.9 Kg/m^2^ Healthy control group, 20-40 years old	Compared to the control group, the experimental group showed a significant increase in the α-diversity of the gut microbiota and significant changes in the abundance of specific flora, which were not associated with antidiabetic drugs. Among them, P Copri decreases, while *Faecalibacterium praussnitzii* and *Akkermansia* with anti-inflammatory effects increase.	The intervention group also had significantly lower blood glucose, total and LDL cholesterol, FFA, HbA1c, triglycerides and area under the CRP curve, and increased antioxidant activity compared to the control group.
Jian et al. (16) Finland and 8 other countries	Low energy diet for the first 8 weeks and weight maintenance for the last 148 weeks (*n* = 211)	Low-carbohydrate, low-fat	Multicenter randomized controlled trial, 3 years	Prediabetes overweight adult patients, *n* = 211, 25–70 years old, BMI ≥ 25 kg/m^2^	There was a significant increase in the relative abundance of several genera linked to enhanced metabolism. Changes in microbiota composition and predicted function were strongly correlated with weight loss. The initial characteristics of the gut microbiota accounted for approximately 25% of the variability in overall changes in adiposity prior to low-energy diet treatment.	Subjects lost an average of 11.5% of body weight and 22% of total body fat during the intervention, with significant improvements in all metabolic parameters. 76 subjects returned to normal blood glucose levels. Substantial interindividual variability was observed in the changes induced by the low-energy diet in variables related to glucose metabolism and total body fat.
Ismael et al. (17) Portugal	Mediterranean diet (*n* = 9)	High fiber and unsaturated fat	Single-arm trial, 12 weeks	T2DM, *n* = 9 (6 males, 3 females), 40–80 years old (mean 66 ± 9 years), except for 1 subject, all received oral hypoglycemic drugs	After 4 weeks, there was an increase in the abundance of intestinal bacteria, and the ratio of *Prevotella*/*Bacteroides* also increased. Bacterial diversity showed a negative correlation with HbA1c, while bacterial abundance exhibited negative correlations with FBS and HOMA-IR. Changes in gut microbiota seemed to precede alterations in a conventional biomarker for type 2 diabetes, namely HbA1c.	HbA1c and HOMA-IR were significantly reduced after 12 weeks. Blood lipid profiles showed no concomitant changes. Alkaline phosphatase activity (a marker of intestinal inflammation and permeability) in fecal samples was negatively correlated with HbA1c and positively correlated with bacterial diversity.
Deledda et al. (18) Italy	Experimental group: ketogenic diet (*n* = 6) Control group: Mediterranean diet (*n* = 5)	Very low-carb, high-fat	Randomized controlled trial, 12 weeks	T2DM newly diagnosed and without complications, *n* = 11 (6 males, 5 females), 45-65 years, BMI ≥ 28 Kg/m^2^	In the ketogenic diet group, there was a significant increase in beneficial microbiota groups, along with a decrease in microbiota groups associated with obesity (*Firmicutes* and Actinobacteriota) or other diseases. The Mediterranean diet group exhibited a significant increase in *Actinobacteria* and *Firmicutes*.	The beneficial effects of the ketogenic diet on anthropometric parameters were more significant than those of the Mediterranean diet, but there were no statistically significant differences in biochemical improvements. Macrogenomic alterations associated with certain metabolic pathways were found only in the ketogenic diet group.
Ren M, et al. (19) China,	Experimental group: almond-based low-carbohydrate diet a-LCD (*n* = 22) Control group: low-fat diet LFD (*n* = 23)	Low-carb, high-fat	Randomized controlled trial, 12 weeks	T2DM, *n* = 45, ≥18 years old	The consumption of a low-calorie diet (a-LCD) notably augmented the presence of short-chain fatty acid-producing bacteria, including *Roseburia, Ruminococcus*, and *Eubacterium*.	HbA1c levels were significantly lower during the study period in both groups compared to baseline. At Month 3, the a-LCD group had higher GLP-1 concentrations than the LFD group, had a greater decrease in HbA1c levels than the LFD, and significantly improved depressive symptoms.
Balfegó et al. (20) Spain	Experimental group: sardine diet (*n* = 19) Control group: general diet recommended for diabetes without sardines (*n* = 16)	High unsaturated fat	Randomized controlled trial, 6 months	T2DM, *n* = 35 (16 males, 19 females), BMI of 26–35 kg/m^2^, 40–70 years old, not receiving insulin and oral hypoglycemic drugs.	Both dietary interventions effectively lowered the concentrations of phylum *Firmicutes* and E. coli compared to their respective baselines. Moreover, the intervention group displayed a reduced *Firmicutes*/*Bacteroidetes* ratio and an increased abundance of *Bacteroides*-*Prevotella*.	There was no significant difference in glycemic control between the groups. Plasma insulin and HOMA-IR were reduced in both groups at 6 months after baseline. Plasma lipocalin increased only in the intervention group (+40.7%) compared to baseline levels. Omega-3 index increased by 2.6% in the experimental group and by 0.6% in the control group.
Karusheva et al. (21) Germany	Experimental group: reduced branched-chain amino acid diet (BCAA-) Control group: complete amino acid diet (BCAA+)	Reduction in branched-chain amino acids	Crossover test, 4 weeks	T2DM, *n* = 12, 40–60 years old, BMI of 28–35 kg/m^2^, disease duration < 5 years.	In comparison to the BCAA+ diet, the BCAA- diet intervention demonstrated an 11% decrease in the abundance of *Firmicutes* and a remarkable 40% increase in the abundance of *Bacteroidetes*.	After the BCAA-diet, insulin secretion was reduced, postprandial insulin sensitivity was increased, and mitochondrial efficiency in adipose tissue was stimulated.
Meleshko et al. (22) Ukraine	Experimental group: personalized diet (*n* = 35) Control group (*n* = 21)	NA	Randomized controlled trial, 18 days	T2DM, *n* = 56, 39–68 years old, all female.	Enterococcus faecalis, Escherichia coli, lac+, and Candida spp. significantly decreased, while *Lactobacillus* spp. significantly increased.	Significant improvements in blood glucose, lipid profile (cholesterol, LDL, HDL, VLDL, triglycerides) and inflammatory markers (IL-1 β, IL-10, IgA, TNF-α).
Shoer et al. (23) Israel	Experimental group: personalized diet (*n* = 100) Control group: Mediterranean diet (*n* = 100)	NA	Randomized controlled trial, 6 months	Prediabetes, *n* = 200, adults	The personalized diet had a greater effect on the gut microbiota than the Mediterranean diet. The personalized diet resulted in a significant increase in the relative abundance and alpha diversity of 19 gut microbiota species. flavonifractor plautii, Roseburia hominis, Ruthenibacterium lactatiformans and Faecalibacterium prausnitzii increased significantly in abundance. The Mediterranean diet resulted in a significant increase in the relative abundance of four gut microbiota species.	Compared to the Mediterranean diet, the personalized diet had a greater effect on glycemic control (HbA1c).

FBS, fasting blood sugar; HbA1c, glycated hemoglobin; HOMA-IR, homeostasis model assessment of insulin resistance; FFA, free fatty acids; plasma CRP, C-reactive protein; IL-6, interleukin; Functional food diet: Rich in soluble fiber, prebiotics, plant protein, and n-3 unsaturated fatty acids; Personalized diet: Using developed algorithms, personalized diets are selected based on the patient's gut microbiota, immune, and biochemical parameters.

The authors apologize for this error and state that this does not change the scientific conclusions of the article in any way. The original article has been updated.

