# Supplementary material for: Dietary intervention improves metabolic levels in patients with type 2 diabetes through the gut microbiota: a systematic review and meta-analysis
Source: Front Nutr. 2024 Jan 8;10:1243095. doi: 10.3389/fnut.2023.1243095 (PMC10800606; doi:10.3389/fnut.2023.1243095)
Supplement: Supplementary file 1 [file Table_1.DOCX]

Attachment Table S1: Nutrient intake and expected nutrient intake of each experimental group after dietary intervention.

| Author | Dietary  patterns | Energy (kcal/day) | | Carbohydrate (%) | | Protein (%) | | Lipids (%) | | Fiber (g/day) | |
| --- | --- | --- | --- | --- | --- | --- | --- | --- | --- | --- | --- |
|  |  | Baseline | After  intervention | Baseline | After  intervention | Baseline | After  intervention | Baseline | After  intervention | Baseline | After  intervention |
| Candela | Ma-Pi 2 diet | 1964-1988 | 1803 | 45.3-45.6 | 73 | 18.2-19.3 | 11.8 | 35.4-36.2 | 15.2 | 10.3-10.8 | 29 |
| Zhao | High fiber diet | 1924.93±129.67 | 1874.87±71.1 | 52.03±2.16 | 56.58±1.09 | 16.94±0.63 | 15.88±0.49 | 31.03±1.86 | 27.54±1.07 | 12.12±1.24 | 37.10±1.90 *** |
| Chen | High fiber diet | NR | NR | NR | NR | NR | NR | NR | NR | NR | 17.9 |
| Medina | Functional food diet | NR | NR | NR | （45-55） | NR | （15-20） | NR | （25-30） | NR | （20-35） |
| Jian | Low energy diet | NR | （810） | NR | （41） | NR | （44） | NR | （15） | NR | （13.3） |
| Ismael | Mediterranean diet | 1849.57±662.42 | 1778.49±475.07 | 49.97±13.12 | 59.56±6.43 | 22.82±8.80 | 20.00±5.84 | 9.27±4.64 | 19.38±6.87* | NR | NR |
| Deledda | Mediterranean diet | 1516±367 | 1161±206 | 42.5±3.1 | 34.7±5.2 | 19.7±2.1 | 26.7±2.6* | 38.8±2.9 | 40±4.3 | 13.8±6.7 | 15.5±5.0 |
|  | Ketogenic diet | 1840±281 | 1630±104 | 42.0±8.6 | 45.8±4.1 | 19.6±2.9 | 19.0±2.6 | 37.8±8.1 | 35.2±2.3 | 20.8±5.8 | 22.8±7.6 |
| Ren | Low-carbon diet | 1686.34±231.25 | 1642.08±227.74 | NR | 40 | NR | 22 | NR | 38 | NR | NR |
|  | Low-fat diet | 1781.91±280.91 | 1764.77±297.40 | NR | 59 | NR | 16 | NR | 25 | NR | NR |
| Balfegó | Sardine diet | 1829.5±129.9 | 1626.2±89.2* | NR | NR | NR | NR | 44.4±1.9 | 43.8±0.8 | 15.5±1.3 | 17.3±1.2 |
| Karusheva | BCAA+ and BCAA- diet | NR | NR | NR | （55） | NR | （15） | NR | （30） | NR | NR |
| Meleshko | Personalized diet | NR | NR | NR | NR | NR | NR | NR | NR | NR | NR |
| Shoer | Personalized diet | NR | NR | NR | NR | NR | NR | NR | NR | NR | NR |

Data are expressed as mean or mean ± SD. * indicates p ≤ 0.05 compared to baseline, ** indicates p ≤ 0.01 compared to baseline, *** indicates p ≤ 0.001 compared to baseline, NR: Not reported Not reported. values in parentheses are actual post-intervention intakes not reported, but expected intakes of experimental design nutrients are reported.

Attachment Table S2. Partial significant changes in metabolic pathways after dietary intervention.

| Intervention measures | Increased metabolic pathways | Reduced metabolic pathways |
| --- | --- | --- |
| ketogenic diet | Cell.motility.and.secretion, Protein.folding.and.associated.processing, Carotenoid.biosynthesis, Fructose.and.mannose.metabolism, Lipoic.acid.metabolism, Base.excision.repair, Biosynthesis.of.vancomycin.group.antibiotics, Photosynthesis.proteins, Bisphenol.degradation, Insulin.signaling.pathway, Linoleic.acid.metabolism, Lysosome, Photosynthesis, Porphyrin.and.chlorophyll.metabolism, Porphyrin.and.chlorophyll.metapglism, Secretion. system, Biosynthesis.of.siderophore.group.nonribosomal.peptides, Prenyltransferases, Steroid.biosynthesis, Lysine.degradation, Non.homologous.end.joining, Valine..leucine.and.isoleucine.degradation, Membrane.and.intracellular.structural.molecules, Bacterial.motility.proteins, Citrate.cycle..TCA.cycle, Riboflavin.metabolism, RNA.degradation, Amino.acid.metabolism, Drug.metabolism.cytochrome.P450, Metabolism.of.xenobiotics.by.cytochrome.P450, alpha.Linolenic.acid.metabolism, Flagellar.assembly, Glycosphingolipid.biosynthesis.ganglio.series, Toluene.degradation, Two.component.system, Folate.biosynthesis, Other.glycan.degradation, Retinol.metabolism, Nucleotide.metabolism, Glycosaminoglycan.degradation, Ubiquinone.and.other.terpenoid.quinone.biosynthesis | Methane.metabolism, Cytoskeleton.proteins, Energy.metabolism, Peptidases, Naphthalene.degradation, Translation.factors, Chromosome, Histidine. Metabolism, Restriction.enzyme, Carbohydrate.digestion.and.absorption, Ethylbenzene.degradation, Penicillin.and.cephalosporin.biosynthesis, Limonene.and.pinene.degradation, Transcription.factors, Carbon.fixation.in.photosynthetic.organisms, Phenylalanine.metabolism, Histidine.metabolism, General.function.prediction.only, Other.transporters, Cell.cycle.Caulobacter, Glycan.biosynthesis.and.metabolism, D.Glutamine.and.D.glutamate.metabolism, Others, Benzoate.degradation, Ribosome.Biogenesis, Sporulation, Xylene.degradation, Polyketide.sugar.unit.biosynthesis, Lipid.biosynthesis.proteins, Geraniol.degradation |
| Low energy diet | Propanoate.metabolism, Lysine.biosynthesis, Lipoic.acid.metabolism, Vitamin.B6.metabolism, Glycine.serine.and.threonine.metabolism, Valine.leucine.and.isoleucine.degradation, Fatty.acid.degradation, Glycosaminoglycan.degradation | Nitrogen.metabolism, Sulfur.metabolism, N.Glycan.biosynthesis, C5.Branched.dibasic.acid.metabolism, Flagellar.assembly |
| fiber-rich longevity Ma-Pi 2 diet | D-glutamine metabolism, D-Glutamate metabolism, Unsaturated fatty acid biosynthesis, Taurine metabolism, cysteine metabolism, methionine metabolism, valine metabolism, leucine metabolism, isoleucine metabolism | Alanine metabolism, Arachidonic acid metabolism, Polyketose biosynthesis, Oxidative phosphorylation, Biosynthesis of glycosphingolipids |

Attachment Figure S1: Risk of bias for the included studies.


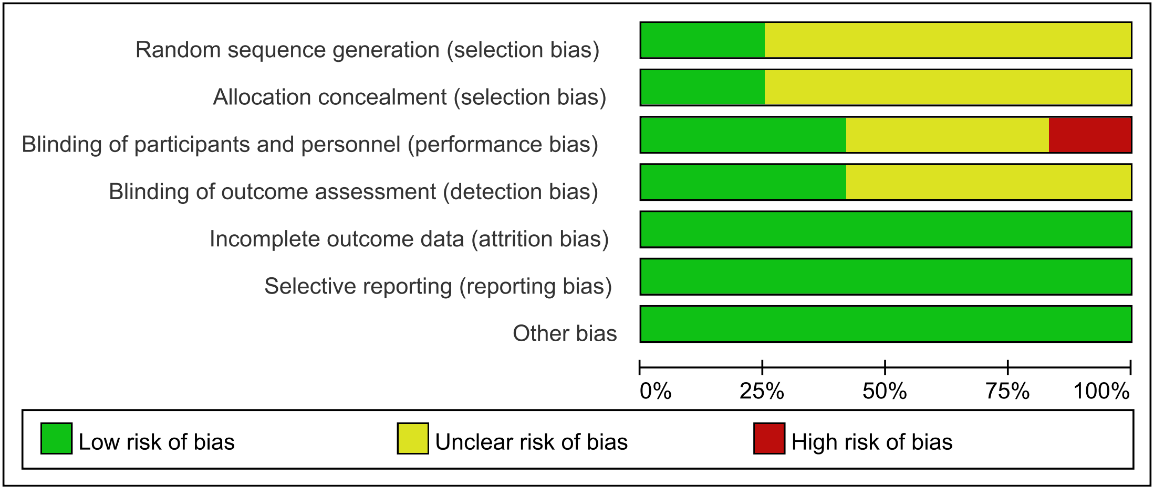


(a) Risk of bias graph for the included studies.


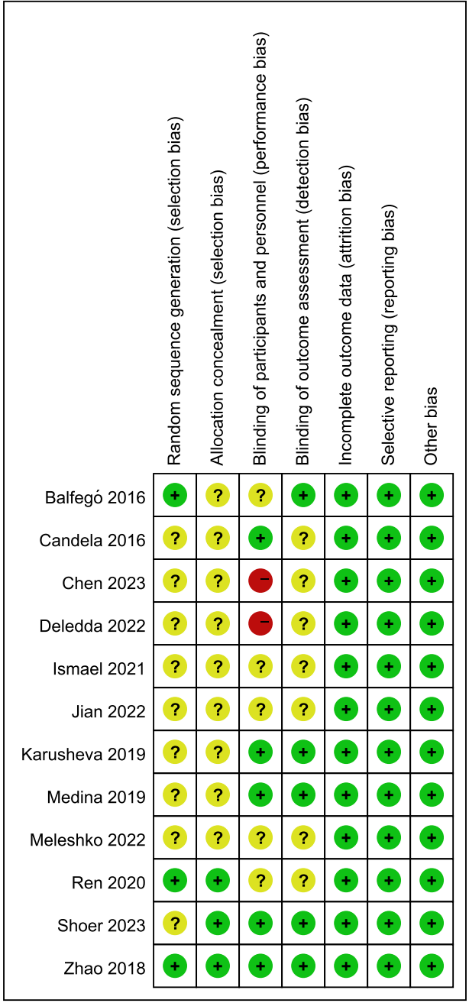


(b) Summary of risk of bias for included studies.

Attachment Figure S2: Summary of Meta Analysis Results.

1.1 Blood glucose metabolism.


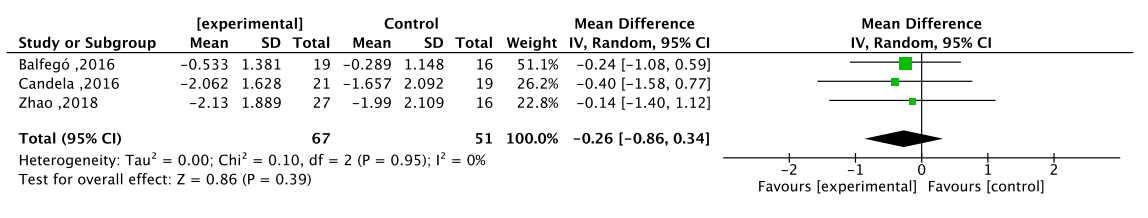


(A) Compared with the recommended diet for type 2 diabetes patients, the effect of diet intervention on fasting blood glucose (FBG) in the experimental group.


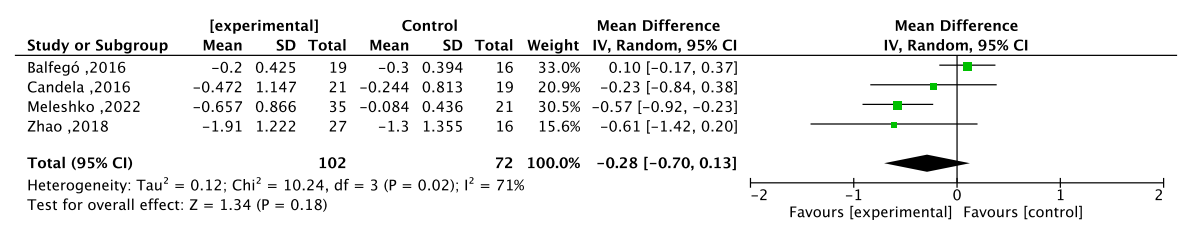


1. Compared with the recommended diet for type 2 diabetes patients, the effect of dietary intervention on HbA1c in the experimental group.


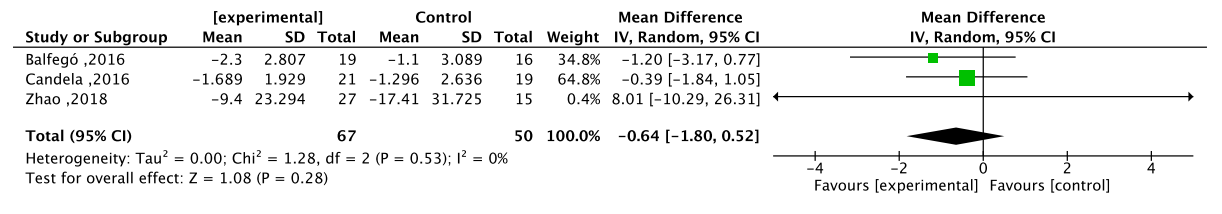


1. Compared with the recommended diet for patients with type 2 diabetes, the effect of dietary intervention on homeostasis model assessment of insulin resistance (HOMA-IR) in the experimental group.


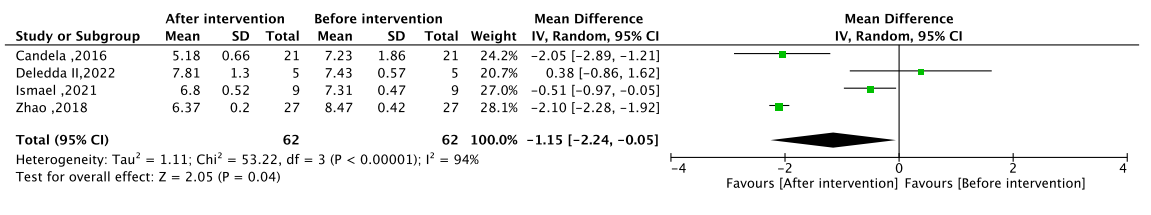


1. Self control study on the effect of high dietary fiber diet intervention on fasting blood glucose (FBG) (mmol/L).


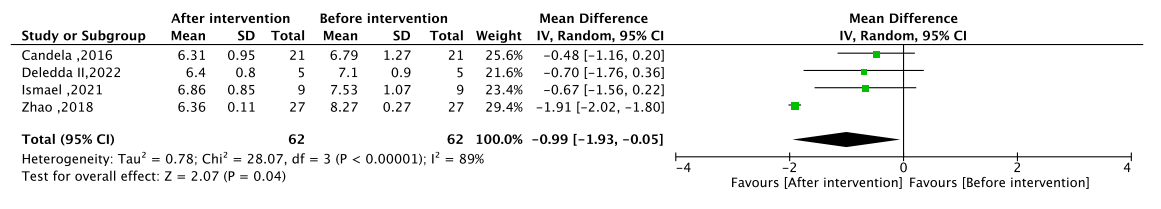


1. Self control study on the effect of high dietary fiber diet intervention on glycated hemoglobin (HbA1c) (%).


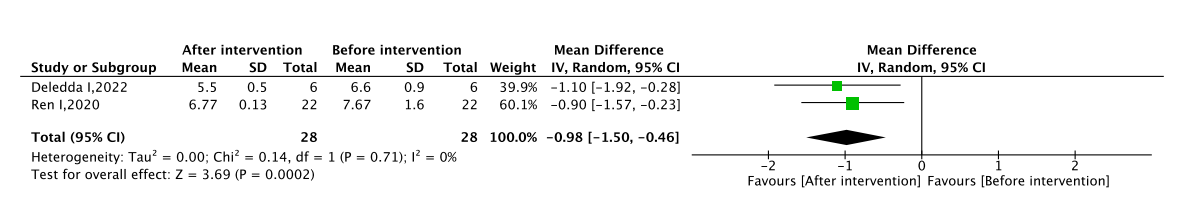


1. Self control study on the effect of high-fat and low-carbon water diet intervention on (HbA1c) (%).

1.2 Lipid metabolism.


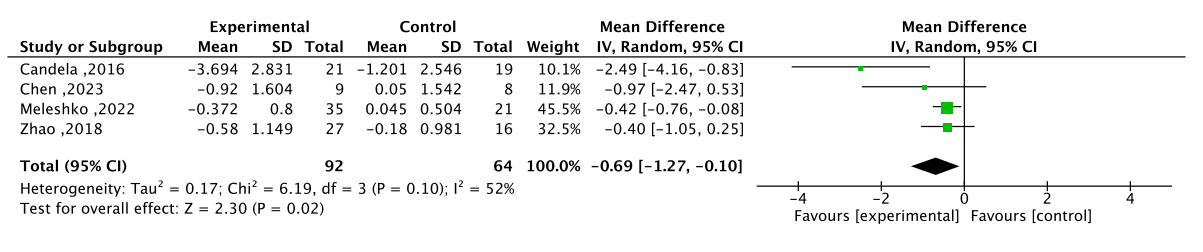


1. Compared with the recommended diet for type 2 diabetes patients, the effect of dietary intervention on total cholesterol (mmol/L) in the experimental group.


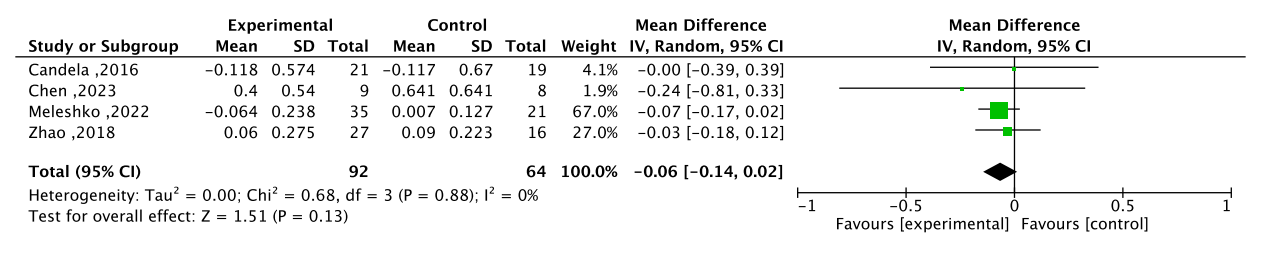


(H) Compared with the recommended diet for type 2 diabetes patients, the effect of dietary intervention on high-density lipoprotein cholesterol (HDL-C) (mmol/L) in the experimental group.


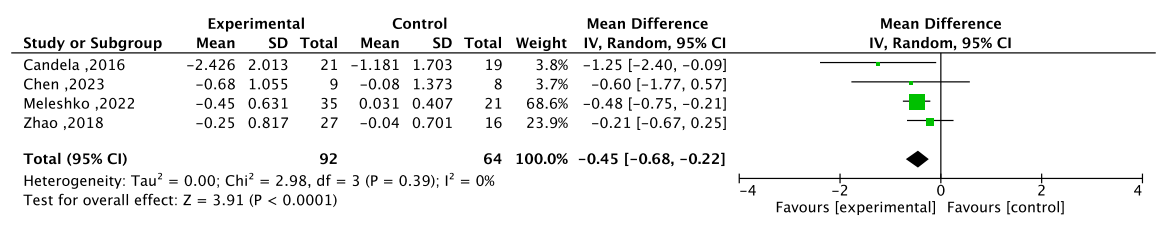


(I) Compared with the recommended diet for type 2 diabetes patients, the effect of dietary intervention on low-density lipoprotein cholesterol (LDL-C) (mmol/L) in the experimental group.


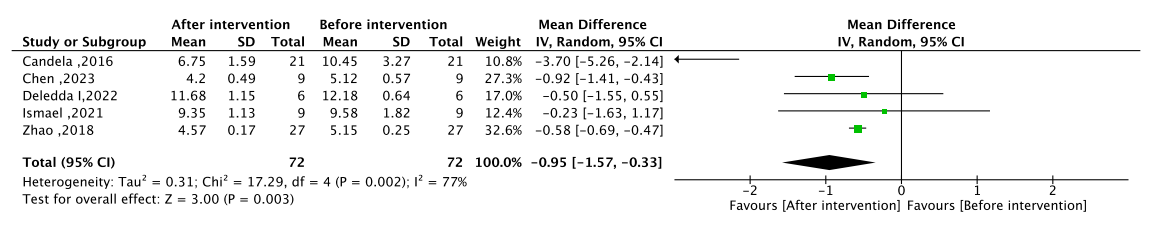


1. Self control study on the effect of high dietary fiber diet intervention on total cholesterol (mmol/L).


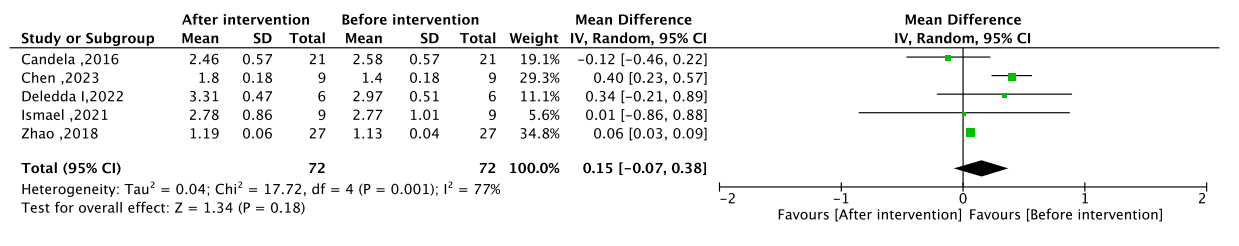


1. Self control study on the effect of high dietary fiber diet intervention on high-density lipoprotein cholesterol (HDL-C) (mmol/L).

1.3 BMI


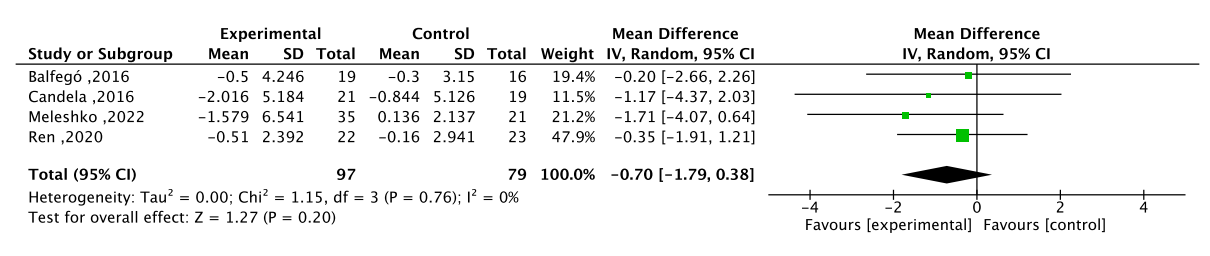


1. Compared with the recommended diet for type 2 diabetes patients, the effect of diet intervention on body mass index (BMI) in the experimental group.
